# In silico investigation of critical binding pattern in SARS-CoV-2 spike protein with angiotensin-converting enzyme 2

**DOI:** 10.1038/s41598-021-86380-2

**Published:** 2021-03-25

**Authors:** Farzaneh Jafary, Sepideh Jafari, Mohamad Reza Ganjalikhany

**Affiliations:** 1 Core Research Facilities (CRF), Isfahan University of Medical Science, Isfahan, Iran; 2 Department of Cell and Molecular Biology, Faculty of Biological Science and Technology, University of Isfahan, Isfahan, Iran

**Keywords:** Biochemistry, Computational biology and bioinformatics

## Abstract

Severe acute respiratory syndrome coronavirus-2 (SARS-CoV-2) is a newly-discovered coronavirus and responsible for the spread of coronavirus disease 2019 (COVID-19). SARS-CoV-2 infected millions of people in the world and immediately became a pandemic in March 2020. SARS-CoV-2 belongs to the beta-coronavirus genus of the large family of Coronaviridae. It is now known that its surface spike glycoprotein binds to the angiotensin-converting enzyme-2 (ACE2), which is expressed on the lung epithelial cells, mediates the fusion of the cellular and viral membranes, and facilitates the entry of viral genome to the host cell. Therefore, blocking the virus-cell interaction could be a potential target for the prevention of viral infection. The binding of SARS-CoV-2 to ACE2 is a protein–protein interaction, and so, analyzing the structure of the spike glycoprotein of SARS-CoV-2 and its underlying mechanism to bind the host cell receptor would be useful for the management and treatment of COVID-19. In this study, we performed comparative in silico studies to deeply understand the structural and functional details of the interaction between the spike glycoprotein of SARS-CoV-2 and its cognate cellular receptor ACE2. According to our results, the affinity of the ACE2 receptor for SARS-CoV-2 was higher than SARS-CoV. According to the free energy decomposition of the spike glycoprotein-ACE2 complex, we found critical points in three areas which are responsible for the increased binding affinity of SARS-CoV-2 compared with SARS-CoV. These mutations occurred at the receptor-binding domain of the spike glycoprotein that play an essential role in the increasing the affinity of coronavirus to ACE2. For instance, mutations Pro462Ala and Leu472Phe resulted in the altered binding energy from − 2 kcal mol^−1^ in SARS-COV to − 6 kcal mol^−1^ in SARS-COV-2. The results demonstrated that some mutations in the receptor-binding motif could be considered as a hot-point for designing potential drugs to inhibit the interaction between the spike glycoprotein and ACE2.

## Introduction

Coronaviruses belong to the family of Coronaviridae and cause different disorders in poultry and mammals^1^. In humans, they cause mild respiratory tract infection, such as some cases of the common cold, Middle East Respiratory Syndrome (MERS), Severe acute respiratory syndrome (SARS), and coronavirus disease 2019 (COVID-19)^2^. Severe acute respiratory syndrome coronavirus (SARS-CoV) was initially reported in Guangdong, China, in 2002^3^. The human-to-human transmission of SARS-CoV infection, leading to the outbreak in 2003 with about a case fatality rate (CFR) of 10%^4^; however, Middle-East respiratory syndrome coronavirus (MERS-CoV) was reported in Saudi Arabia in June 2012^5^. MERS-CoV indicated a CFR of almost 34.4%, even with its limited human-to-human transmission^4^. In December 2019, 2019-nCoV was primarily reported in Wuhan, China, in patients with pneumonia, and its transmission rate exceeded the infection rate of both SARS-CoV and MERS-CoV in humans^2^.

Coronavirus structure, consisting of the viral genomic RNA and nucleocapsid (N) phosphoproteins, which is surrounded by a lipid bilayer. The RNA genome of the virus contains about 30,000 nucleotides and produces 3 or 4 types of structural proteins, including membrane (M), spike (S), and envelope proteins (E), as well as the hemagglutinin-esterase (HE) protein, which is detected in some types of coronaviruses^6^. By producing adequate copy numbers of novel structural proteins and genomic RNA, the particles are assembled. The assembly and release of virions are the final steps of the viral life cycle.

The spike glycoprotein (also called protein S) has a key role in viral attachment, entry, and fusion and serves as a target for developing vaccines, antibodies and other potential inhibitors such as peptides for inhibition of viral attachment to the cells^7^. The protein S is produced as a precursor that contains almost 1,300 residues; then cleaved to two subunits; 1- a carboxyl (C)-terminal (S2 subunit) region and an amino (N)-terminal (S1 subunit) region. A trimer spike is synthesized and exposed on the viral envelope by assembling three S1/S2 heterodimers. The S1 subunit contains a receptor-binding domain which mediates the viral entry into the host cells through binding to the host receptor. Also, the S2 subunit contains two heptad repeat areas that participate in the fusion process^8^.

The spike glycoprotein has 1273 residues consisting of five regions, including receptor-binding domain (RBD) (residues 319–541), receptor-binding motif (RBM) that binds to human ACE2 (residues 437–508), fusion peptide (residues 788–806), heptad repeat-1 (residues 920–970) and heptad repeat-2 (residues 1163–1202). This construct plays critical roles in viral entry to host cells and, consequently, viral infection^9^.

It has been demonstrated that different receptors are bounded to RBDs of MERS-CoV and SARS-CoV^10^. Angiotensin-converting enzyme 2 (ACE2) is one of the important receptors that can bind to SARS-CoV; while, dipeptidyl peptidase-4 binds to MERS-CoV. ACE2 is an enzyme with 805 residues that is expressed on the surface of the cell membrane of several tissues (lungs, arteries, heart, kidney, and intestine) and interacts with the spike glycoprotein in some coronaviruses, including HCoV-NL63, SARS-CoV, and SARS-CoV-2^11,12^. ACE2 has two domains in the extracellular region, including the zinc metallopeptidase domain (residues 19–611) and the C-terminal domain (residues 612–740). The zinc metallopeptidase domain is composed of three regions that interacts with SARS-CoV spike glycoprotein through residues positioned at 30–41, 82–84, and 353–357^13^.

ACE2 also binds to SARS-CoV-2 through the S protein expressed on the surface of the virus. Thus, it is vital to deeply investigate the RBD expressing on SARS-CoV-2 S as a potential target for developing the potent inhibitors, designing the vaccines, and production of neutralizing antibodies^14^. The binding affinity of ACE2-spike is crucial for SARS-CoV-2 infection efficiency and completely dependent to the structure and interaction pattern of spike glycoprotein form SARS-CoV-2. Since the binding domain from each structures is available, so it is possible to measure the affinity of the whole complex^15^.

Herein, we investigated the interaction pattern of ACE2-Spike protein complex using in silico approaches to deeply understand the mechanism underlying the virus attachment and explain any potential differences in the binding pattern of SARS-CoV-2 and SARS-CoV to their cognate receptors to be able to propose new promising drugs with the highest efficiency for COVID-19 treatment. It seems that the inhibition of Spike-ACE2 interaction is the most straightforward and efficient method for the prevention of SARS-CoV-2 infection using peptides or small molecules. Previously, we have performed computational methods to understand the protein–protein interaction pattern at atomic levels to seek potentially specific peptide inhibitors for cancer treatment^16,17^. In this study, several computational methods were utilized, including molecular dynamics simulation^18^, MM-PBSA^19^ and interaction pattern analyses. The detailed molecular events could be explained by these methods from conformational alterations upon the interaction of the virus with its receptor to molecular interactions between the viral protein and the related receptors at atomic level. An intensive structural assessment was performed to investigate the interaction between the virus spike protein and its receptor. Three structure forms of ACE2-spike protein complexes, namely 2ajf, 6m0j, as well as a chimeric structure 6vw1 have been selected for the study. The chimeric structure is a receptor-binding domain of SARS-CoV that acts as a scaffold, while the receptor-binding motif of SARS-CoV-2 acts as a functional group for the interaction with ACE2^20^. Therefore, the comparison of three structures could be feasible to understand the role of spike mutations on the binding affinity to ACE2.

## Results

### Analysis of spike (S) glycoprotein-ACE2 receptor interaction

At first, three crystal structures (2ajf, 6m0j and 6vw1) were obtained, and then the differences in their structures and sequences were identified (Figs. S1 and S2). The results indicated that the majority of the mutations occurred in the receptor-binding motif of SARS-CoV-2 compared with SARS-CoV which is listed in the Table S1. This motif plays an essential role in spike-receptor interaction^21^.

#### Interaction patterns analysis

**Figure 1:**
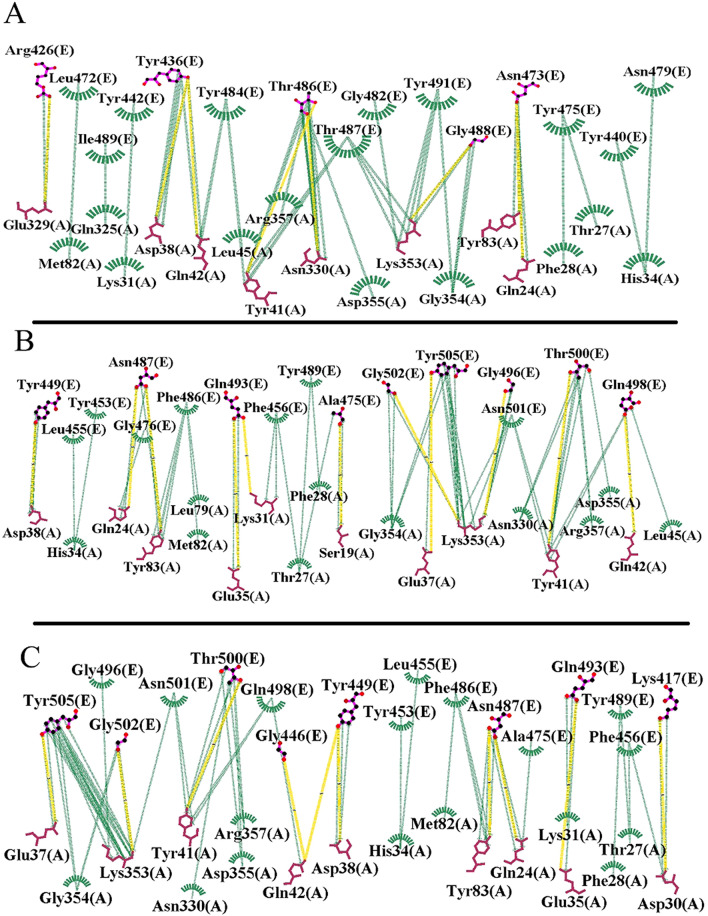
Two-dimensional interaction schemes for the spike-ACE2 complex: SARS-CoV (**A**), chimeric structure (**B**), and SARS-CoV-2 (**C**). ACE2 and spike proteins residues denoted by A and E in parenthesis respectively. Also, hydrogen bonds and hydrophobic interactions are colored in yellow and green lines respectively. The images have been obtained by LigPlot^+^ v.1.4.5 (https://www.ebi.ac.uk/thornton-srv/software/LigPlus/)^22^.

In the second step, hydrophobic, electrostatic, cation-pi interactions and hydrogen bonds formed in the receptor-spike complex were analyzed using Ligplot^+^^22^. Based on the results, four regions in ACE2 interact with specific residue in the spike glycoprotein of SARS-CoV that summerized in Table 1. Also, a group of residues in the SARS-CoV-2 spike protein are present in the interface region of the four regions in ACE2. Comparative analysis of the RBD between SARS-CoV-2 and SARS-CoV showed the presence of several mutations in this area including Tyr442Leu, Leu443Phe, Leu472Phe, Asn479Gln, Tyr484Gln, and Thr487Asn. These mutations could be considered as important players in the binding sites of the spike protein in the ACE2 receptor, and could cause interaction pattern alterations between SARS-CoV-2 and ACE2. For instance, mutation Tyr442Leu leads to change in interaction pattern between spike-ACE2 from SARS-CoV to SARS-CoV-2. By means that, instead of residue 31, residue 34 of ACE2 is involved in virus-receptor interaction in the binding site which we will further discuss about this area. Accordingly, other mutations can also cause changes in the interacting pattern of SARS-CoV-2-ACE2 when compared with SARS-CoV. The detailed interaction patterns are shown in Fig. 1.

**Table 1:** The interaction list for SARS-CoV-ACE2 and SARS-CoV2-ACE2 complexes obtained from Ligplot^+^.

Complex	Receptor residues	Spike residues
SARS-CoV-ACE2	(24–38, 41–42–45, 82–83 and 330–357)	Arg426, Tyr436, Tyr440, Tyr442, Leu443, Leu472, Asn473, Tyr475, Asn479, Gly482, Tyr484, Thr486 Thr487, Gly488, Ile489
SARS-CoV2-ACE2	(24–38, 41–42–45, 82–83 and 330–357)	Lys417, Gly446, Tyr449, Tyr453, Leu455, Phe456, Ala475, Phe486, Asn487, Tyr489, Gln493, Gly496, Gln498, Thr500, Asn501, Gly502, Tyr505

#### Contact area analysis spike-ACE2 complex during MD simulation

PyContact^23^ was used for the analysis of non-covalent interactions between the spike and ACE2 receptor during the Molecular Dynamics (MD) simulation^18^ to characterize the critical areas in ACE2 involved in the interaction with the spike. The contact areas between SARS-CoV and SARS-CoV-2 with ACE2 receptor were depicted in Figs. S3 and S4. The obtained results showed 5 and 6 regions of ACE2 interacted with SARS-CoV and CoV-2-ACE2 respectively and the highest contact area in both complexes were detected in residues 35–194 and 266–344. These two regions are not constantly involved in the interaction, but instead residues 35–50 and 300–330 have the highest participation in the interaction in ACE2. Also, analysis of the interface area demonstrated that a group of critical residues including residues 353–358 from region 346–372 of the ACE2 receptor interacted with the SARS-CoV-2 spike protein, while such interaction was not found in the SARS-CoV-ACE2 complex.

On the other hand, the interface region of the chimeric structure is completely different from two other structures (Figure S5). Based on our results, there are seven regions in ACE2 that interact with chimeric structure. The chimeric structure has an interface region including 11 residues (495–505), which were non-specific for protein–protein interaction in the virus-receptor complex. In residues 19–33, the interface region has higher contact area than SARS-CoV-2 but lower than SARS-CoV. In three regions of ACE2 (residues 16–175, residues 224–246, residues 248–326), the interface area was about 700 Å^2^, while in two other regions (347–373 and 542–615) was about 300 Å^2^. Such a difference may stem from the integration of two regions from two different proteins that cause nonspecific interactions.

#### Hydrogen bond analysis

**Figure 2:**
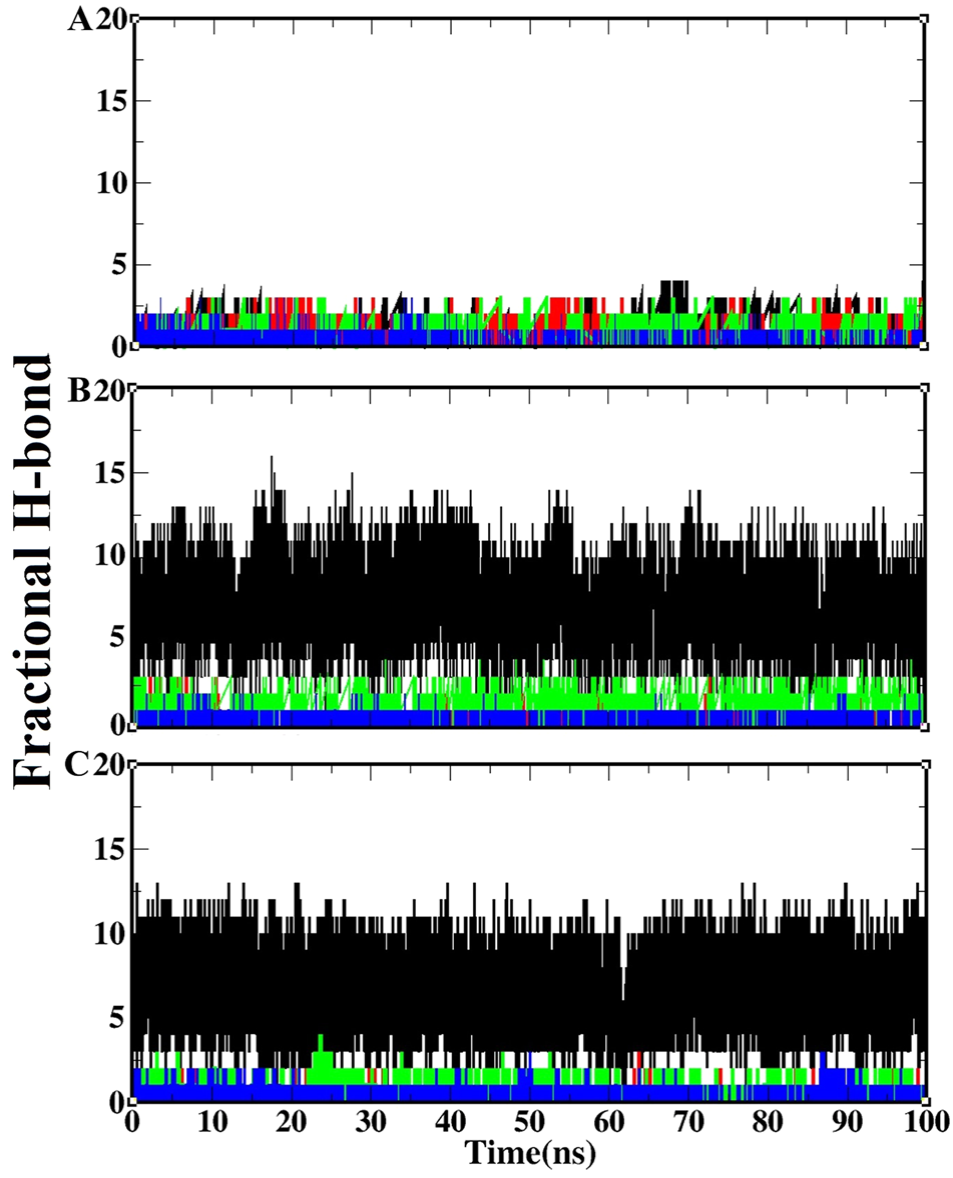
The fractional H-bonds of the receptor-spike protein complex during the simulation: hydrogen bonds between four regions of the ACE2 receptor (19–33, 35–54, 325–331, and 334–339) and receptor-binding motif in SARS-CoV (**A**), chimeric structure (**B**) and SARS-CoV-2 (**C**). Black, red, green and blue lines in each graph are related to residues 19–33, 35–54, 353–358 and 325–331 of the ACE2 receptor, respectively. The fractional H-bond graphs have been plotted using *xmgrace* from Grace plotting tool (https://plasma-gate.weizmann.ac.il/Grace/).

Hydrogen bonds were analyzed in the four regions of the ACE2 receptor (19–33, 35–54, 325–331, and 334–339) and receptor-binding motif of the three spike structures during the simulation, and they are summarized in Fig. 2. Obtained results showed that the four regions of the ACE2 receptor (19–33, 35–54, 353–358, and 325–331) participated in the interaction with the receptor-binding motif of the spike protein from SARS-CoV and SARS-CoV-2.

The number of H-bonds in residues 19–33 of the receptor when interacting with the receptor-binding motif of the spike during the simulation was higher in complexes of chimeric structure and SARS-CoV-2 than that of SARS-CoV. Also, the number of H-bonds in residues 334–339 of the receptor in SARS-CoV-2 and chimeric structure complexes were more than that of SARS-CoV. However, the number of H-bonds in the spike-receptor complex in two regions 35–54 and 325–331 of SARS-CoV-2 and chimeric structure complexes were lower than SARS-CoV. In this way, the number of H-bonds in the four regions of the SARS-CoV-ACE2 complex when interacting with the receptor-binding motif of the spike protein was not the same as SARS-CoV-2 and chimeric structure complexes. Therefore, it appears that the number of H-bonds in the receptor-binding motif of SARS-CoV-2 when interacting with ACE2 was more than SARS-CoV. These four regions of the receptor interact with specific parts of the virus receptor-binding motif, which we will discuss later.

#### Interaction network analysis

**Figure 3:**
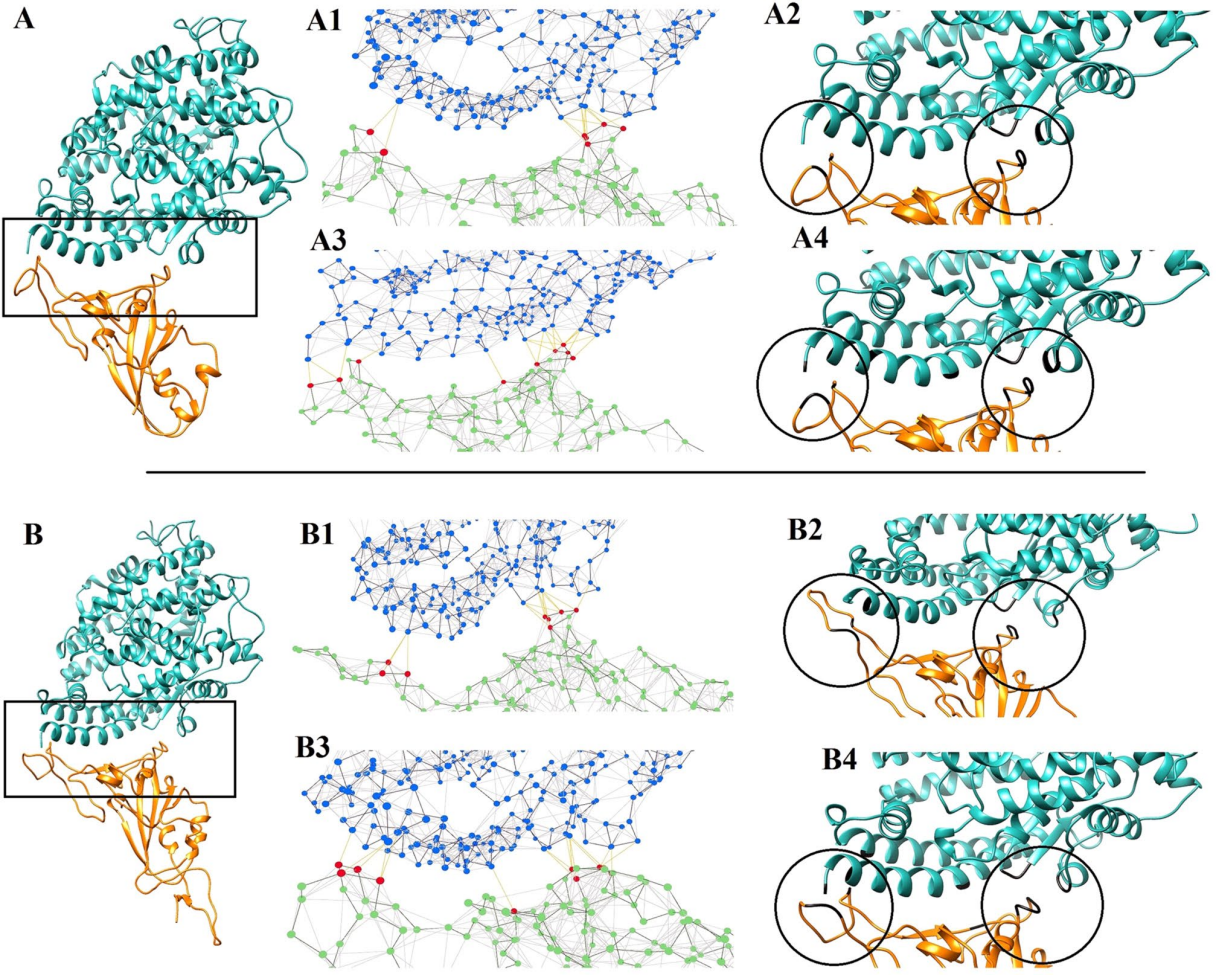
The interaction network analysis of ACE2-spike protein complex during the simulation: (**A**) in SARS-CoV (2ajf) and (**B**) SARS-CoV-2 (6m0j) structures. The PDB structures obtained from the initial (A1 and A2 for SARS-CoV, and B1 and B2 for SARS-CoV-2) and final (A3 and A4 for SARS-CoV, and B3 and B4 for SARS-CoV-2) 500 frames of the simulation that were analyzed by NAPS. The red points in A1, A3, B1 and B3 indicate critical amino acids that participated in the spike-ACE2 interaction The images have been obtained by UCSF Chimera 1.13.1 (http://www.rbvi.ucsf.edu/chimera/)^47^ and NAPS server (http://bioinf.iiit.ac.in/NAPS/)^24^.

The interactions pattern between the spike glycoprotein and ACE2 has been evaluated for SARS-CoV and SARS-CoV-2 using Network Analysis of Protein Structures (NAPS)^24^. The structures were obtained from the initial and final 500 frames of the simulation. Our results showed that the spike is attached to the receptor through two regions at the beginning and end of the receptor-binding motif during the simulation. Although other interactions also were occurred by the end of the simulation, it seems that these two areas play an important role in the attachment of the virus spike to its cognate receptor (Fig. 3).

### Binding free energies analysis

#### Analysis of binding free energies for three forms of the spike-receptor complex

The molecular mechanics Poisson–Boltzmann surface area (MM-PBSA)^19^ method was utilized for the calculation of the binding energies of SARS-CoV, SARS-CoV-2, and the chimeric structure when bound to ACE2 (Table 2). The lowest binding energy was related to SARS-CoV-2 with − 31.5759 ± 2.4425 kcal mol^−1^. According to the results, electrostatic interactions have an essential role in binding affinities between SARS-CoV-2 and its receptor.

**Table 2:** The MM-PBSA binding energies (kcal·mol^−1^) for the three spike-receptor structures.

	Δ*G-*bind	ENPOLAR	EPB	EEL	VDWAALS
SARS-CoV	− 12.0104 ± 3.0752	− 62.4930	167.6406	− 162.4268	− 83.3501
SARS-CoV-2	− 31.5759 ± 2.4425	− 71.5213	179.2997	− 181.2610	− 100.9902
Chimeric	− 13.4810 ± 2.6388	− 64.2094	139.3610	− 134.3633	− 86.1871

The binding free energy for the spike-receptor was the same for chimeric and SARS-CoV complexes (− 13.4810 ± 2.6388 kcal mol^−1^ and − 12.0104 ± 3.0752 kcal mol^−1^ respectively).

It seems that some of the mutations in the receptor-binding motif might have an essential role in the increased affinity of SARS-CoV-2 to ACE2; however, the impact of mutations on the other regions of RBD is not great as much as mutations effects on RBM. According to the above results, interaction mechanism of SARS-CoV and SARS-CoV-2 spike with ACE2 receptor has been investigated in details and roles of mutations in changing the SARS-CoV-2 affinity for ACE2 have been also assessed.

#### Binding free energy decomposition for spike-receptor complexes

**Figure 4:**
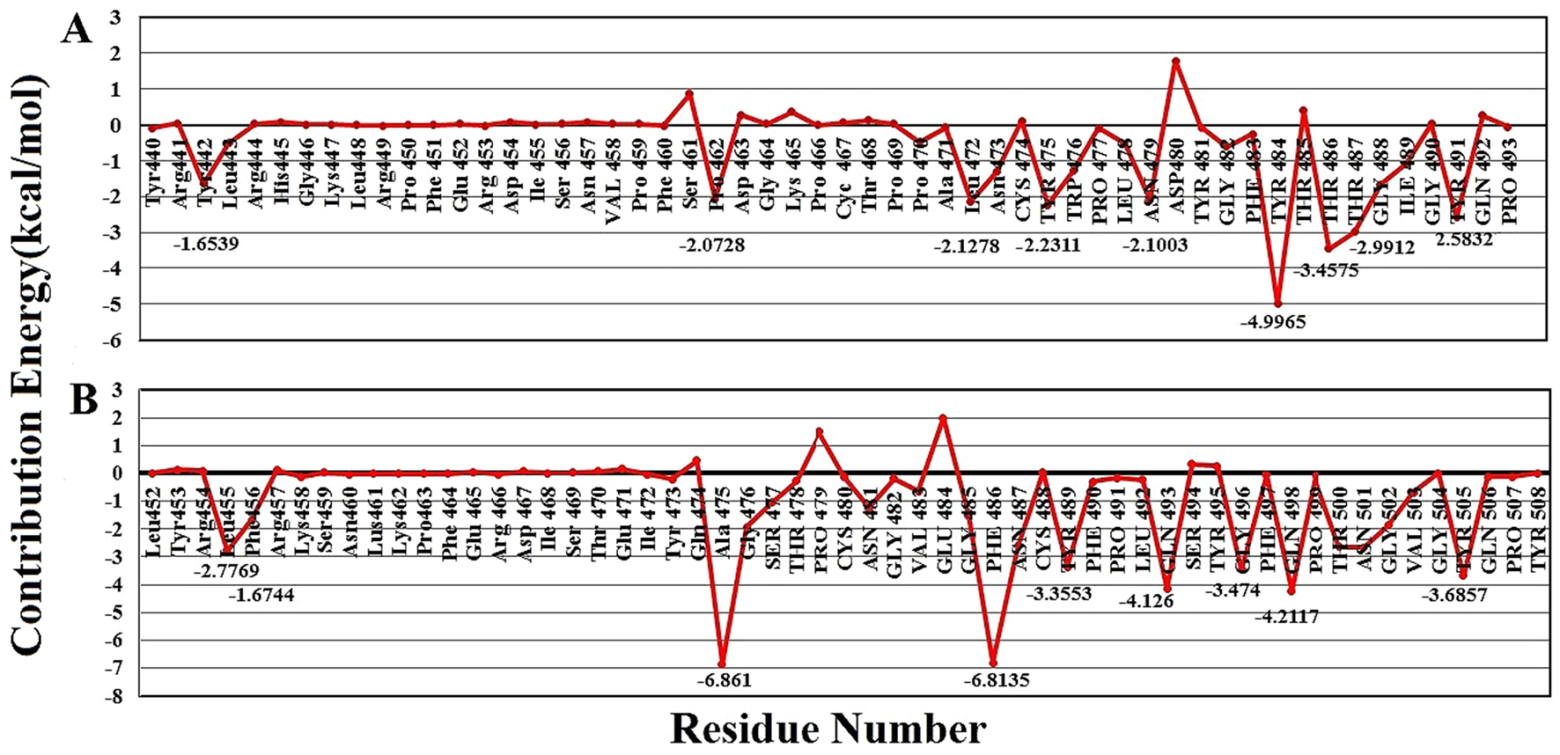
Free energy decomposition of the spike protein residues in the spike-ACE2 complex: (**A**) SARS-COV and (**B**) SARS-COV-2. The red graph shows binding free energy for each residue in the receptor-binding motif of the spike protein.

The analysis of free energy decomposition was performed on the spike-ACE2 complexes. The results are depicted in Fig. 4 and Tables S2 and S3. Free energy decomposition analysis helps to find contribution of a single residues by summing its interactions over the entire residues.

These results revealed that two mutation including Tyr442Leu and Leu443Phe in SARS-CoV-2 have changed binding free energies from − 1.6539 ± 0.4785 kcal·mol^−1^ and − 0.5149 ± 0.0363 kcal·mol^−1^ in SARS-CoV to − 2.7769 ± 0.17222 kcal·mol^−1^ and − 1.6744 ± 0.2814 kcal·mol^−1^ in SARS-CoV-2.

Mutations Pro462Ala and Leu472Phe in SARS-CoV-2 altered the binding free energy from − 2 kcal·mol^−1^ in SARS-CoV to − 6 kcal·mol^−1^. These two residues are located at the beginning and end of a loop that interacts with the N-terminal domain of the receptor. The flexibility of this loop might facilitate the binding of the spike protein to its receptor which is shown in figure S6. The Pro462Ala mutation makes the region flexible as a hinge, and therefore, facilitates the binding of the virus to its receptor.

Mutation Asn479Gln also altered the binding free energy of these from − 2 kcal·mol^−1^ in SARS-CoV to − 4 kcal·mol^−1^ in SARS-CoV-2. Therefore, it seems that this region also plays a critical role in the interaction of coronavirus to ACE2. This finding was also confirmed by our native contact results which will be discussed in the next section.

**Figure 5:**
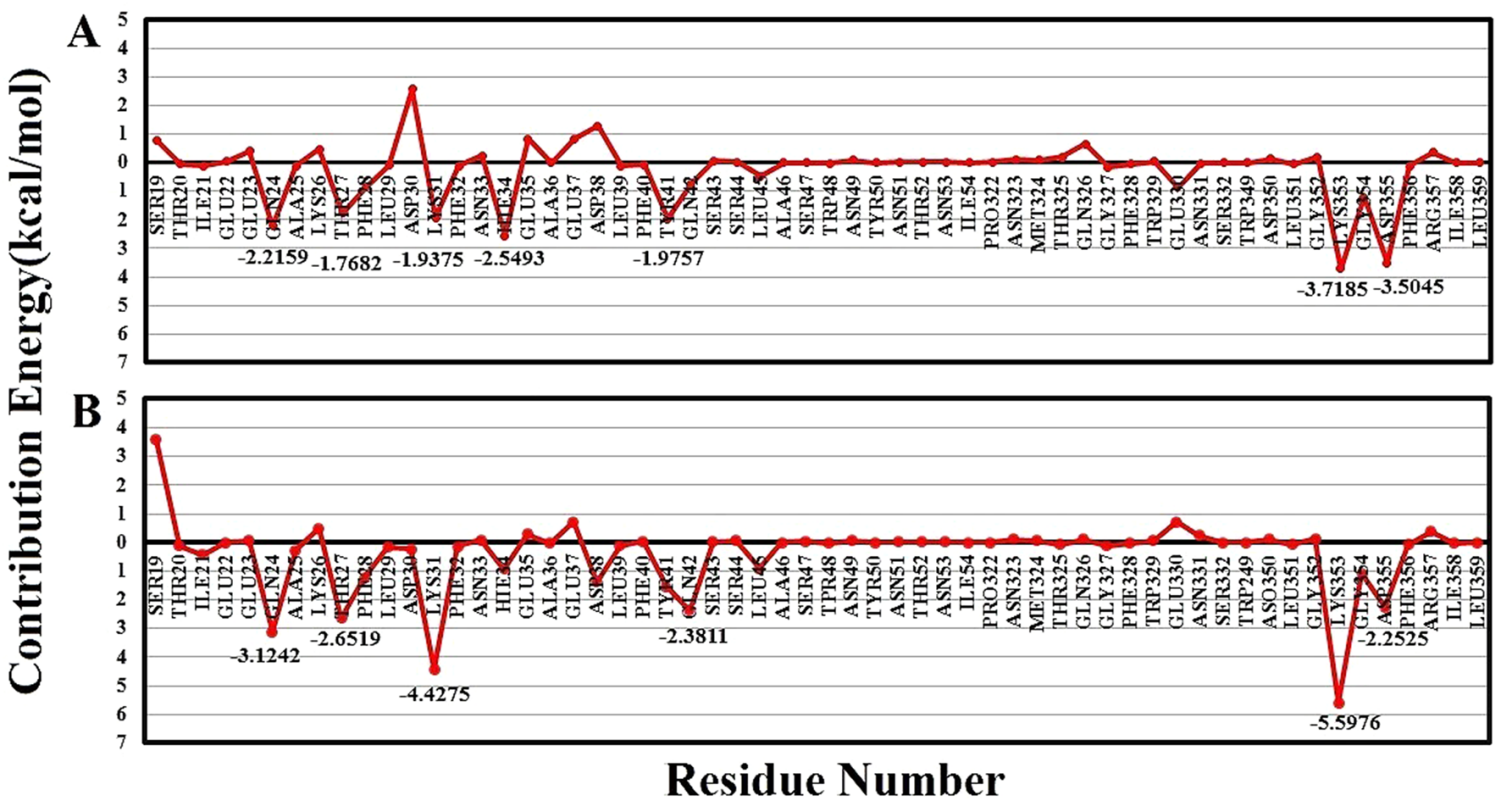
Free energy decomposition of the ACE2 residues in the spike-ACE2 complex: (**A**) SARS-COV and (**B**) SARS-COV-2. The red graph shows binding free energy for each residue in ACE2 receptor.

Also, binding free energy decompositions for ACE2 residues have been calculated, and the results are shown in Fig. 5. According to the results, free energy contributions of Lys31 and Lys353 are significantly different between SARS-CoV and SARS-CoV-2. The changes in free energy contribution of Lys31 from − 1.9 kcal·mol^−1^ in SARS-CoV to − 4.4 kcal·mol^−1^ in SARS-CoV-2 and Lys353 from − 3.7 kcal·mol^−1^ in SARS-CoV to − 5.5 kcal·mol^−1^ in SARS-CoV-2 could be related to the different interacting residues form spike protein and the mutations occurring in this region. The results demonstrated that Lys31 has a fundamental role in the interaction of ACE2 with N-terminal and middle regions of the receptor-binding motif. However, residue Lys353 contributes in the interaction with almost the end of the receptor-binding motif.

### Contact patterns of the SARS-CoV and SARS-CoV-2 interactions with ACE2

**Figure 6:**
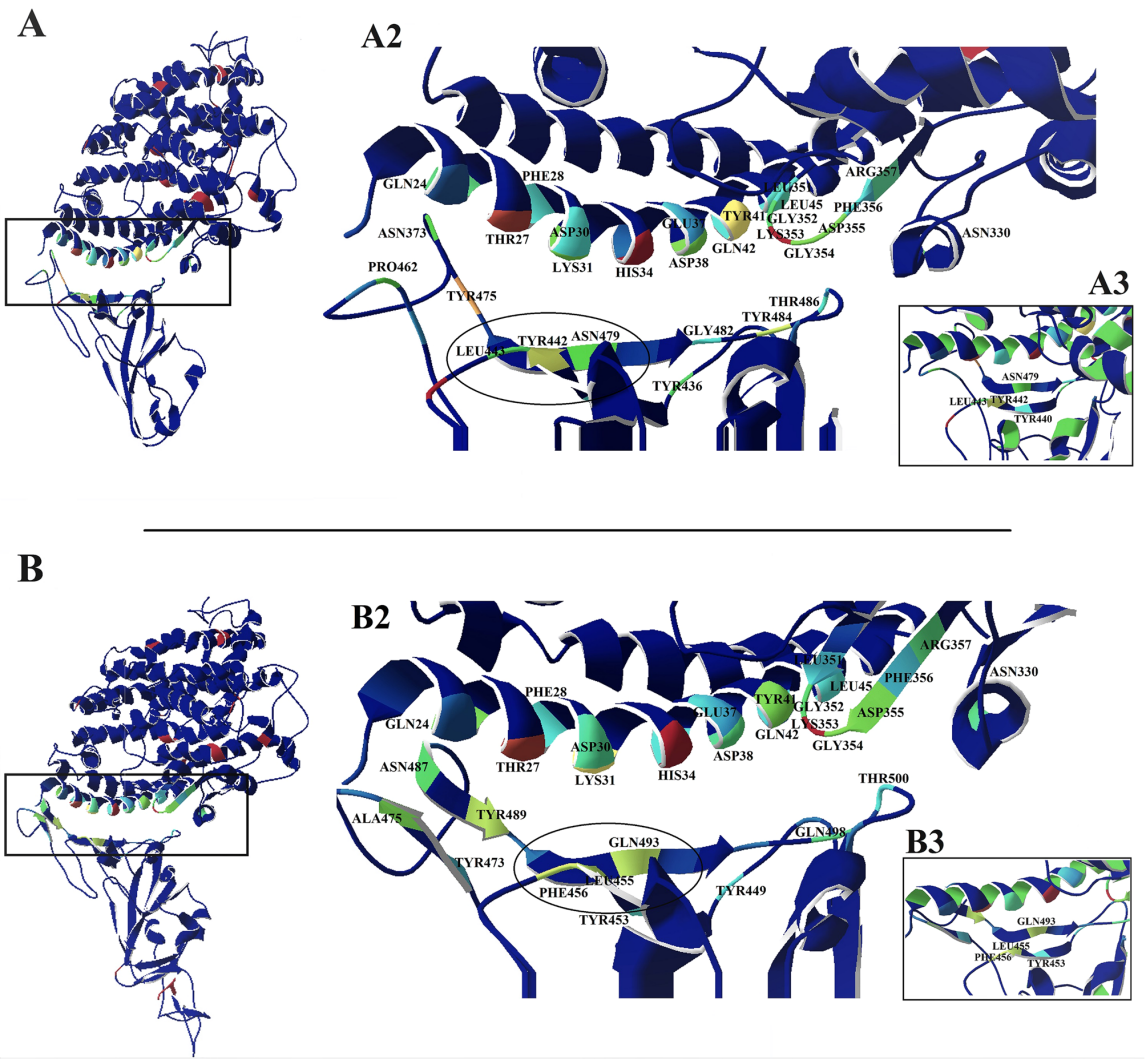
Native contact pattern for the SARS-CoV and SARS-CoV2 in interaction with ACE2: The native contact pattern between the receptor-binding motif of the spike protein and ACE2 in SARS-CoV (**A**) and SARS-CoV-2 (**B**) during the simulation. Residues with high values of the native contact (based on R-factor) involved in the interactions are designated as green, yellow, and red which identified in part A2 and B2. A3 and B3 are the region located in the middle of the receptor-binding motif in SARS-CoV and SARS-CoV-2 respectively that acts as a clamp for the binding of the virus to ACE2. The images have been obtained by Swiss-PDB viewer 4.0.1^46^ (https://spdbv.vital-it.ch/).

For more details about the interaction between spike-ACE2 and also, to confirm the significance of the mutations that have occurred in the structure of SARS-CoV-2 compared with SARS-CoV, the native contact pattern^25^ was analyzed in two structures of SARS-CoV and SARS-CoV-2 during the simulation. The native contact pattern between the receptor-binding motif of the spike protein and ACE2 is shown in Fig. 6 and Table 3.

**Table 3:** The native contact interaction list for SARS-CoV-ACE2 and SARS-CoV-2-ACE2.

Complex	Receptor residues	Spike residues
SARS-CoV-ACE2	Gln24, Thr27, Phe28, Asp30, Lys31, His34, Glu37, Asp38, Tyr41, Gln42, Leu45, Leu351, Gly352, Lys353,Gly354, Asp355, Phe356, Arg357, Asn330	Thr436, Tyr440, Tyr442, Leu443, Pro462, Asn373, Thr475, Asn479, Gly482, Tyr484, Thr486
SARS-CoV2-ACE2	Gln24, Thr27, Phe28, Asp30, Lys31, His34, Glu37, Asp38, Tyr41, Gln42, Leu45, Leu351, Gly352, Lys353, Gly354, Asp355, Phe356, Arg357, Asn330	Tyr449, Tyr453, Leu455, Phe456, Tyr473, Ala475, Asn487, Tyr489, Gln493, Gln498, Thr500

The maximum number of native contacts in the interaction between the receptor-binding motif of the spike protein and ACE2 receptor was observed in residues 31, 353 of ACE2 for both structures (SARS-CoV and SARS-CoV-2). These two residues are considered as hotspot points that interact with the beginning and terminal regions of the receptor-binding motif in the spike protein.

In addition to regions at the beginning and terminal of the receptor-binding motif, another region in the middle of the receptor-binding motif was also involved in the interaction between the spike protein and ACE2 receptor. This region acts as a clamp in the binding of the virus to ACE2 (Fig. 6A3,B3). Indeed, in this region is including Tyr440, Tyr442, Leu443, and Asn479 of SARS-CoV as well as Tyr453, Phe455, Leu456 and Gln493 of SARS-CoV-2, create a cavity that, through its two edges interacts with Thr27, Asp30, Lys31, His34 and Glu35 of ACE2.

There are three mutations in this region, including Tyr442Lue, Leu443Phe, and Asn479Gln.

These mutations cause an increase in the binding affinity of the ACE2 receptor to SARS-CoV-2 compared with SARS-CoV. For more confidence, selected alanine scanning was done for residues (Tyr440, Tyr442, Leu443, and Asn479 for SARS-CoV and Tyr453, Leu455, Phe456, Gln493 for SARS-CoV-2) and MM-PBSA method was used in order to calculate the binding affinity of each substitution (Tables 4 and 5). According to the result, altering each of these residues lead to reduce the binding affinity in SARS-CoV-2 and the lowest binding affinity was observed for Phe456Ala substitution.

**Table 4:** The MM-PBSA binding energies (kcal·mol^−1^) for selected alanine scanning in residue Tyr440, Tyr442, Leu443, and Asn479 for SARS-CoV.

	Δ*G-*bind	ENPOLAR	EPB	EEL	VDWAALS
SARS-CoV	− 12.0104 ± 3.0752	− 62.4930	167.6406	− 162.4268	− 83.3501
Tyr440Ala	− 15.66 ± 1.797	− 59.467	207.317	− 205.933	− 89.190
Tyr442Ala	− 17.022 ± 1.486	− 66.721	212.750	− 208.356	− 91.047
Leu443Ala	− 7.662 ± 3.104	− 58.622	200.357	− 192.209	− 86.263
Asn479Ala	− 17.278 ± 2.933	− 55.405	204.127	− 206.330	− 76.317
Ensemble	− 10.248 ± 2.591	− 52.137	200.049	− 199.123	− 74.067

**Table 5:** The MM-PBSA binding energies (kcal·mol^−1^) for selected alanine scanning in residue Tyr453, Leu455, Phe456, Gln493 for SARS-CoV-2.

	Δ*G-*bind	ENPOLAR	EPB	EEL	VDWAALS
SARS-CoV-2	− 31.5759 ± 2.4425	− 71.5213	179.2997	− 181.2610	− 100.9902
Tyr453Ala	− 24.695 ± 2.035	− 67.607	180.057	− 180.548	− 91.102
Leu455Ala	− 25.640 ± 0.895	− 68.387	188.118	− 188.790	− 91.327
Phe456Ala	− 18.018 ± 1.56	− 67.424	172.313	− 171.792	− 88.939
Gln493Ala	− 25.183 ± 3.134	− 69.044	176.134	− 174.301	− 97.341
Ensemble	− 16.926 ± 2.779	− 59.912	170.744	− 172.889	− 81.089

In contrast to the selected SARS-CoV-2 alanine scanning results, except for Leu443Ala, the rest of substitution for SARS-CoV have increased the binding affinity to ACE2.

For more details on how these four residues can change the binding pattern and affinity of protein–protein interaction between spike and receptor, 2D interaction pattern analysis was done for SARS-CoV-ACE2 and SARS-CoV-2-ACE2 complexes and then compared with alanine mutated complexes **(**figure S7 and S8). The structures were obtained from the final 50 frames of the simulation. According to the results, substitution of Tyr440, Tyr442, Leu443, and Asn479 to alanine in of SARS-CoV, lead to change in interaction pattern and also binding affinity of this complex. For instance, substitution of Leu443 alanine decreased the number of interaction between two proteins in which the lowest binding affinity was also observed in this substitution. Similar results were observed in SARS-CoV-2-ACE2 complex. Comparatively, by replacing Tyr453, Leu455, Phe456, Gln493 with alanine in SARS-CoV-2, the interaction pattern and type of bonds have been altered. For instance, Gln493 interacts with Lys31, His34 and Glu35 but alanine substitution eliminates Lys31 and Glu35 interactions. Similarly, Phe456 interacts with Asp30 and Thr27 but alanine substitution eliminates Thr27 interactions and the lowest binding affinity is observed for this substitution.

Therefore, these mutations not only altered the interaction pattern but also deceased the binding affinity which was very significant for SARS-CoV-2. As reported in the MMPBSA results, substitution of these residues with to alanine increased the binding energy in the SARS-CoV and SARS-CoV-2 from − 12.0104 kcal mol^−1^ and − 31.5759 kcal mol^−1^ to − 10.248 kcal mol^−1^ and − 16.926 kcal mol^−1^ respectively.

## Discussion

Virus-receptor recognition is a primary viral infection phase and plays a decisive role in tissue tropism in host cells. The improved binding affinity of SARS-CoV for ACE2 has been correlated with the disease severity and virus transmissibility in humans^26–29^. The epidemiological studies indicated that the infectivity of SARS-CoV isolated from three epidemics are between 2002 and 2003 was higher and more pathogenic in humans than the isolates of the re-emergence era between 2003 2004. According to some reports, specific mutations in the spike glycoprotein may influence the binding affinity of SARS-CoV to ACE2^28,30,31^. In some studies, it was reported that SARS-CoV-2 employs ACE2 as an entry receptor. It was also suggested that the SARS-CoV-2-ACE2 complex has the same affinity as the SARS-CoV-ACE2 complex isolated during 2002–2003^32^. Another study reported that the binding affinity between ACE2 and the RBDs of SARS-CoV-2 and SARS-CoV has similar ranges^33,34^. This indicates that it could be generalized to humans, as many types of SARS-CoV-2 could be transmitted from human-to-human. On the other hand, some studies reported that the binding affinity of the S-protein to the ACE2 receptor is 20 folds higher than that of SARS-CoV, as confirmed by Cryo-EM analysis of the spike protein structure in the perfusion conformation^35^.

In this study, we employed in silico methods to investigate the interaction of the spike protein of SARS-CoV and SARS-CoV-2 with the ACE2 receptor in atomic details to understand the biological process by which the virus infects the host cells. Therefore, by analysis of the detailed interaction patterns, we will able to discover new methods to neutralize virus infection. Herein, the spike-receptor interaction analyses were performed to find hotspot residues involved in such feasible interactions. According to protein–protein interaction results, residues 24–38, 41–42, 82–83, and 330–357 from ACE2 have an important role in interactions of SARS-CoV and SARS-CoV-2 the ACE2 receptor and these results were in line with the findings of Han et al.^36^.

Pycontact was applied to explore the interface regions between ACE2 and spike during the simulation. According to our results, N-terminal domain which includes residues 35–54 and 300–330 of ACE2 in regions 35–194 and 266–344, showed the highest interface area in SARS-CoV-2 and SARS-CoV complexes; however, the interface area between the SARS-CoV-2 and ACE2 in regions 35–194 increases during the simulation and also was higher than of SARS-CoV. The results also demonstrated an interface region including residues 346–372 between SARS-CoV-2 and ACE2 while no such interface region was observed between SARS-CoV and ACE2. This may be due to the fewer interactions in this area in the SARS-CoV-ACE2 complex.

After investigating the binding regions on ACE2, we focused on the H-bond patterns to understand the detailed affinity between the spike glycoprotein and ACE2 receptor in four areas separately (19–33, 35–54, 353–358, and 325–331). According to our results, the highest number of hydrogen bonds was observed in the N-terminal region including residues 353–358 in ACE2 when interacting with the receptor-binding motif of the spike protein in SARS-CoV-2 and chimer structures, which were different with SARS-CoV. Maximum number of H-bonds for SARS-CoV-ACE2 complex was observed between the receptor-binding motif of spike and the residues located at 35–54 and 325–331 from ACE2. The numbers of H-bonds in other regions in SARS-CoV-ACE2 complex were less than SARS-CoV-2 and chimeric structures. The higher number of H-bonds in the receptor-binding motif of SARS-CoV-2 when interacting with ACE2 could explain the higher affinity of SARS-CoV-2 to ACE2. Based on the results, the interaction networks were analyzed for SARS-CoV and SARS-CoV-2 complexes, to discover the interaction pattern between the receptor-binding motif and ACE2.

According to NAPS results, two regions at the beginning and end of the receptor-binding motif may play critical roles in the interaction of the virus to their receptor. Esther et al. also showed there are two loops in RBD of SARS-CoV-2 and SARS-CoV (loop I: residues 474–489, loop II: residues 498–505 for SARS-CoV-2 and loop I: residues 462–472, loop II: residues 484- 491 for SARS-CoV), which interact with the N-terminal helix domain of ACE2 located on both terminal regions^37^.

This result was also confirmed by the MM-PBSA method, indicating that the highest binding affinity of SARS-CoV2 to ACE2 is two folds higher than other complexes. The analysis of MM-PBSA showed that electrostatic interactions have the most significant role in the interaction between the virus and ACE2 in three forms of complexes. This finding was in agreement with other studies. Nguyen et al. reported that the binding affinity of SARS-CoV-2 to ACE2 is a twofold higher, and the binding affinity of both viruses to ACE2 is driven by electrostatic interactions^38^. Consistent with our results, Wrapp et al. indicated that the binding affinity of SARS-CoV-2 to ACE-2 is ~ 10- to 20 folds higher than SARS-CoV^35^.

These results are probably due to mutations in the receptor-binding motif of SARS-CoV-2, resulting in an increase in the affinity to ACE2 when compared with SARS-CoV. Of note, mutations in other regions of RBD influence the interaction of SARS-CoV-2 with ACE2 but not as much as mutations occurring in RBM.

For more details, the free energy decomposition for all residues in binding regions of the spike protein and receptor has been analyzed. We observed several mutations in the receptor-binding motif of the spike protein, leading to a change in binding free energy in the SARS-CoV-2-ACE2 complex when compared with SARS-CoV-ACE2.

Mutation Thr487Asn could explain the increment of the H-bond in region 334–339 in SARS-CoV-2 compared with SARS-CoV. Thr487 has an essential role in recognition of the human ACE2 by the SARS-CoV^39^. Ortega and colleagues also reported that mutation Thr487Asn may result in increasing binding affinity of SARS-CoV-2 with its cognate receptor^40^. Mutations Thr487Asn could also be considered as a reason for increasing of the interaction number as well as interface area in region 327–353 of ACE2 when interacting with SARS-CoV-2 compared with SARS-CoV. Other mutations including Pro462Ala and Leu472Phe are located almost at the beginning and end of the loop region (residues 475–486). Also, mutation Asn479Gln located in the middle part of the receptor-binding motif of the spike protein, leading to a decrease in the binding free energy at this point in SARS-CoV-2 compared with SARS-CoV.

These mutations (Tyr442Leu, Leu443Phe, Pro462Ala, Leu472Phe, Asn479Gln and Thr487Asn) interact with Lys31 and Lys353 of ACE2 that were previously introduced as hotspot points in the spike-ACE2 interaction^18,41^. The analysis of free energy decomposition of ACE2 indicated that the binding free energies of these residues (Lys31 and Lys353) were decreased from SARS-CoV to SARS-CoV-2. The decrease in free binding energy stems from the presence of four mutations that cause an increase in the affinity of SARS-CoV-2 to ACE2. Therefore, mutations Thr487Asn, Pro462Ala, Leu472Phe, and Asn479Gln could be considered as hotspot points in RBD of SARS-CoV2 and play an important role in the interaction of the virus with the two ends of the N-terminal domains of ACE2, leading to higher affinity of SARS-CoV-2 spike protein to its receptor compared with SARS-CoV.

In last step, native contact analysis was used to discover the more details from protein–protein interactions between spike glycoproteins and ACE2 receptor. Based on the native contact pattern results, there are three regions in the receptor-binding motif which involve in spike-ACE2 interaction, including the beginning and end of the receptor-binding motif, and a region located in the middle area of the receptor-binding motif of the spike protein in SARS-CoV and SARS- CoV-2. The middle area is comprised of two beta-sheets that form a clamp-like structure. It is now known that β-sheets play a critical role in function of proteins, such as ligand binding or target recognition domains^42^, protein–protein interactions (PPIs)^43^, and targets of proteases^44^. According to native contact results, it seems that this region plays a fundamental role in the interaction of coronaviruses with ACE2. Also, in previous studies some of the residues which are located in this area were introduced as important point for virus-spike interaction^40^.

To further confirm the importance this area, we employed selected alanine scanning for some critical residues in this region. Based on these results, altering key residues to alanine in this region lead to an increase of binding free energy in SARS-CoV-2 while the binding free energy has decease for SARS-CoV.

Finally, our results might be helpful for addressing how mutations in the receptor-binding motif play significant roles in increasing the affinity of the virus to its receptor, or other mutations in other points which influence the viral infection. Protein–protein interactions (PPIs) are regularly mediated by distinct PPI domains and could be determined by analyzing the 3D structure of the domains due to the intermolecular interactions between the proteins^45^. While direct mutation in PPI area are important, other mutations in residues located outside of the receptor-binding motif could affect the 3D structure of domains and could consequently change the viral affinity to specific receptors which has been explained for the case of chimer-ACE2 complex.

## Conclusion

In the current study, molecular dynamics simulation and binding details of the spike glycoprotein-ACE2 complex for SARS-CoV, SARS-CoV-2, and chimeric structure have been performed. The present study aimed to understand the differences in the binding mechanism of the virus to its receptor in three complex forms since these structures could be regarded as a target for drug design. Also, there are mutations in specific regions of the receptor-binding motif that may have an important effect on the spike affinity to its specific receptor. Therefore, our findings showed that receptor-binding motif of the protein have an essential role in the interaction of the spike protein with ACE2. Mutations in the receptor-binding motif could be regarded as hotspot points for drug design and the inhibition of the spike-ACE2 interaction.

## Methods

### Sequence and structural analysis of Spike (S) glycoprotein and ACE2 receptor

Crystal structures of the SARS coronavirus spike receptor-binding domain in complex with its receptor (PDB code: 2ajf), SARS-CoV-2 spike receptor-binding domain bound to ACE2 (PDB code: 6m0j), and the structure of 2019-nCoV chimeric receptor-binding domain in complex with its receptor (PDB code: 6vw1) were obtained from Protein Data Bank (https://www.rcsb.org). Then, we performed in silico comparison among these structures to deeply investigate the importance of mutations in the receptor-binding motif (RBM) and or other points of the receptor-binding domain (RBD) as well as the spike affinity to receptors to understand the binding of the virus to ACE2.

The structural analysis was conducted by Swiss-PDB viewer 4.0.1^46^ and UCSF Chimera 1.13.1 (developed by the Resource for Biocomputing, Visualization, and Informatics at the University of California, San Francisco, with support from NIH P41-GM103311)^47^. FASTA format of three structure were extracted from Protein Data Bank and were aligned by ClustalW (https://www.ebi.ac.uk/Tools/msa/clustalo).

### Analysis of intermolecular interactions between the Spike (S) glycoprotein and ACE2 receptor

The structural analysis of coronavirus spike receptor-binding domain in complex with its receptor was carried out on three PDB files (2ajf, 6m0j, and 6vw1) by Swiss-PDB viewer and LigPlot^+^ v.1.4.5^22^ to assess the interaction of the protein S with its cognate receptor^20^.

### Molecular dynamics simulation

Molecular dynamics simulation of complex structures was performed to understand the interaction between the spike glycoprotein and its receptor using AMBER20^48^ and pmemd.cuda GPU code and ff14SB force-field^49^. The complex was neutralized by adding Cl^-^ ions to the structure using the LEaP module. Afterward, the structures were immersed in an octahedral box filled with a 10 Å layer of TIP3P water molecules^49^. Then, the topology and the coordination were saved for the subsequent steps in simulations. The energy minimizing of the solvated spike-ACE2 complex was performed in two phases. First, the ions and water were minimized by 3000 steps; then, the entire system was minimized by 5,000 steps employing the steepest-decent and conjugate gradient algorithms. For calculating non-covalent interactions by the PME, the cutoff distance was adjusted to 10 Å in the periodic boundary condition. The system was heated from 0 to 300 K for 200 ps, with the NVT ensemble using Langevin thermostat with a collision frequency of 2 ps^50^. The bonds were constrained, including hydrogen atoms using the SHAKE algorithm^51^. Prior to the MD production, the equilibration was performed in the NPT ensemble for 1 ns with Berendsen barostat and relaxation time 2 ps, and the pressure was adjusted to 1 atm. Ultimately, MD simulation was performed for 100 ns with the NPT ensemble. The time-step was set at 2 fs, and the coordinates were saved every 0.8 ps.

### Trajectory analysis

The trajectory analysis was carried out using CPPTRAJ^52^ from AmberTools 20 for calculating the fluctuation and native contacts. The fractional H-bond graphs have been plotted using *xmgrace* from Grace plotting tool (https://plasma-gate.weizmann.ac.il/Grace/).

### Interaction analysis of complexes during simulations

PyContact was used as a tool for the analysis of non-covalent interactions from trajectories. PSF (topology) and CDC (trajectory) are used as input formats then the results were expressed as contact score (the number of hydrogen bonds) and solvent accessible surface areas (SASAs) as shown by histograms or contact maps^23^.

### The network analysis of protein structures

NAPS server (http://bioinf.iiit.ac.in/NAPS/) was applied to perform the network analysis of protein structures at different snapshots during the simulation process^24^.

### Molecular mechanics Poisson–Boltzmann surface area (MM-PBSA) calculation

In order to compare the binding affinity of the spike glycoprotein-ACE2 complex for three structures (2ajf, 6m0j, and 6vw1), MM-PBSA calculation was conducted with the previous procedures using Amber 20 for 20 ns using ff99SB force-field. The analysis of binding free energies was performed by *mmpbsa.py*^19^.

## Supplementary Information

Supplementary Information

## References

[1] Payne S (2017). Family coronaviridae. Viruses.

[2] Sahin AR (2020). Novel coronavirus (COVID-19) outbreak: a review of the current literature. EJMO.

[3] Zhong NS (2003). Epidemiology and cause of severe acute respiratory syndrome (SARS) in Guangdong, People's Republic of China, in February, 2003. Lancet.

[4] De Wit E, van Doremalen N, Falzarano D, Munster VJ (2016). SARS and MERS: recent insights into emerging coronaviruses. Nat. Rev. Microbiol..

[5] Zaki AM, van Boheemen S, Bestebroer TM, Osterhaus AD, Fouchier RA (2012). Isolation of a novel coronavirus from a man with pneumonia in Saudi Arabia. N. Engl. J. Med..

[6] de Haan CA, Rottier PJ (2005). Molecular interactions in the assembly of coronaviruses. Adv. Virus Res..

[7] Lissenberg A (2005). Luxury at a cost? Recombinant mouse hepatitis viruses expressing the accessory hem agglutinin esterase protein display reduced fi tness in vitro. J. Virol..

[8] Li F (2016). Structure, function, and evolution of coronavirus spike proteins. Ann. Rev. Virol..

[9] Belouzard S, Millet JK, Licitra BN, Whittaker GR (2012). Mechanisms of coronavirus cell entry mediated by the viral spike protein. Viruses.

[10] Yin Y, Wunderink RG (2018). MERS, SARS and other coronaviruses as causes of pneumonia. Respirology.

[11] Hamming I (2004). Tissue distribution of ACE2 protein, the functional receptor for SARS coronavirus. A first step in understanding SARS pathogenesis. J. Pathol..

[12] Towler P (2004). ACE2 X-ray structures reveal a large hinge-bending motion important for inhibitor binding and catalysis. J. Biol. Chem..

[13] Yan R (2020). Structural basis for the recognition of the SARS-CoV2 by full-length human ACE2. Science.

[14] Towler P (2004). ACE2 X-ray structures reveal a large hinge-bending motion important for inhibitor binding and catalysis. J. Biol. Chem..

[15] Siebenmorgen T, Zacharias M (2020). Computational prediction of protein–protein binding affinities. WIREs Comput. Mol. Sci..

[16] Tavakoli F, Ganjalikhany MR (2019). Structure-based inhibitory peptide design targeting peptide-substrate binding site in EGFR tyrosine kinase. PloS ONE.

[17] Jafary F, Ganjalikhany MR, Moradi A, Hemati M, Jafari S (2019). Novel peptide inhibitors for lactate dehydrogenase a (LDHA): A survey to Inhibit LDHA activity via disruption of protein–protein interaction. Sci. Rep..

[18] Hollingsworth SA, Dror RO (2018). Molecular dynamics simulation for all. Neuron.

[19] Miller BR (2012). MMPBSA.py: an efficient program for end-state free energy calculations. J. Chem. Theory Comput..

[20] Shang J, Ye G, Shi K (2020). Structural basis of receptor recognition by SARS-CoV-2. Nature.

[21] Li F, Li W, Farzan M, Harrison SC (2005). Structure of SARS coronavirus spike receptor-binding domain complexed with receptor. Science.

[22] Laskowski RA, Swindells MB (2011). LigPlot+: multiple ligand-protein interaction diagrams for drug discovery. J. Chem. Inf. Model..

[23] Scheurer M (2018). PyContact: rapid, customizable, and visual analysis of noncovalent interactions in MD simulations. Biophys. J..

[24] Chakrabarty B, Parekh N (2016). NAPS: network analysis of protein structures. Nucl. Acids Res..

[25] Best R, Hummer G, Eaton W (2013). Native contacts determine protein folding mechanisms in atomistic simulations. Proc. Natl. Acad. Sci..

[26] Guan Y (2003). Isolation and characterization of viruses related to the SARS coronavirus from animals in southern China. Science.

[27] Li W (2004). Efficient replication of severe acute respiratory syndrome coronavirus in mouse cells is limited by murine angiotensin-converting enzyme 2. J. Virol..

[28] Li W (2005). Receptor and viral determ**inants** of SARS-coronavirus adaptation to human ACE2. EMBO J..

[29] Wan Y, Shang J, Graham R, Baric RS, Li F (2020). Receptor recognition by novel coronavirus from Wuhan: An analysis based on decade-long structural studies of SARS. J. Virol..

[30] He JF (2004). Chinese SARS molecular epidemiology consortium. Molecular evolution of the SARS coronavirus during the course of the SARS epidemic in China. Science.

[31] Kan B (2005). Molecular evolution analysis and geographic investigation of severe acute respiratory syndrome coronavirus-like virus in palm civets at an animal market and on farms. J. Virol..

[32] Walls AC (2020). Structure, function, and antigenicity of the SARS-CoV2 spike glycoprotein. Cell.

[33] Tian X (2020). Potent binding of 2019 novel coronavirus spike protein by a SARS coronavirus-specific human monoclonal antibody. Emerg. Microbes Infect..

[34] Lan J (2020). Structure of the SARS-CoV2 spike receptor-binding domain bound to the ACE2 receptor. Nature.

[35] Wrapp D (2020). Cryo-EM structure of the 2019-nCoV spike in the prefusion conformation. Science.

[36] Han Y, Král P (2020). Computational design of ACE2-based peptide inhibitors of SARS-CoV-2. ACS Nano.

[37] Brielle ES, Duhovny DS, Linial M (2020). The SARS-CoV2 exerts a distinctive strategy for interacting with the ACE2 human receptor. Viruses.

[38] Nguyen HL (2020). Does SARS-CoV2 bind to human ACE2 more strongly than does SARS-CoV?. J. Phys. Chem. B.

[39] Lu G, Wang Q, Gao GF (2015). Bat-to-human: spike features determining 'host jump' of coronaviruses SARS-CoV, MERS-CoV, and beyond. Trends Microbiol..

[40] Ortega JT, Serrano ML, Pujol FH, Rangel HR (2020). Role of changes in SARS-COV2 spike protein in the interaction with the human ACE2 receptor: in silico analysis. EXCLI J..

[41] Wu K, Peng G, Wilken M, Geraghty RJ, Li F (2012). Mechanisms of host receptor adaptation by severe acute respiratory syndrome coronavirus. J. Biol. Chem..

[42] Browne JP, Strom M, Martin SR, Bayley PM (1997). The role ofbeta-sheet interactions in domain stability, folding, and target recognition reactions of calmodulin. Biochemistry.

[43] Remaut H, Waksman G (2006). Protein–protein interaction through beta-strand addition. Trends Biochem. Sci..

[44] Tyndall JDA, Nall T, Fairlie DP (2005). Proteases universally recognizebeta strands in their active sites. Chem. Rev..

[45] Lim WA (2010). Designing customized cell signaling circuits. Nat. Rev. Mol. Cell Biol..

[46] Guex N, Peitsch MC (1997). SWISS-MODEL and the Swiss-PdbViewer: An environment for comparative protein modeling. Electrophoresis.

[47] Pettersen EF (2004). UCSF Chimera: A visualization system for exploratory research and analysis. J. Comput. Chem..

[48] Case DA (2020). AMBER 2020.

[49] James AM (2015). ff14SB: Improving the accuracy of protein side chain and backbone parameters from ff99SB. J. Chem. Theory Comput..

[50] Loncharich RJ, Brooks BR, Pastor RW (1992). Langevin dynamics of peptides: The frictional dependence of isomerization rates of N-actylananyl-N’-methylamide. Biopolymers.

[51] Ryckaert JP, Ciccotti G, Berendsen HJC (1977). Numerical integration of the Cartesian equations of motion of a system with constraints: Molecular dynamics of n-alkanes. J. Comput. Phys..

[52] Roe DR, Cheatham TE (2013). PTRAJ and CPPTRAJ: Sofware for processing and analysis of molecular dynamics trajectory data. J. Chem. Theory Comput..

