# Supplementary material for: In silico investigation of critical binding pattern in SARS-CoV-2 spike protein with angiotensin-converting enzyme 2
Source: Sci Rep. 2021 Mar 25;11:6927. doi: 10.1038/s41598-021-86380-2 (PMC7994905; doi:10.1038/s41598-021-86380-2)
Supplement: Supplementary file 1 — Supplementary Information [file 41598_2021_86380_MOESM1_ESM.pdf]

In silico investigation of critical binding pattern in SARS-CoV-2 spike protein with angiotensin-converting enzyme 2

Farzaneh Jafary[1], Sepideh Jafari[2], Mohamad Reza Ganjalikhany[2*]

1. Core Research Facilities (CRF), Isfahan University of Medical Science, Isfahan, Iran

2 Department of Cell & Molecular Biology, Faculty of Biological Science and Technology, University of Isfahan, Isfahan, Iran

* Corresponding author: Mohamad Reza Ganjalikhany

Email: m.ganjalikhany@sci.ui.ac.ir

Tel: +98-31-37932250, P.O. Box: 81744

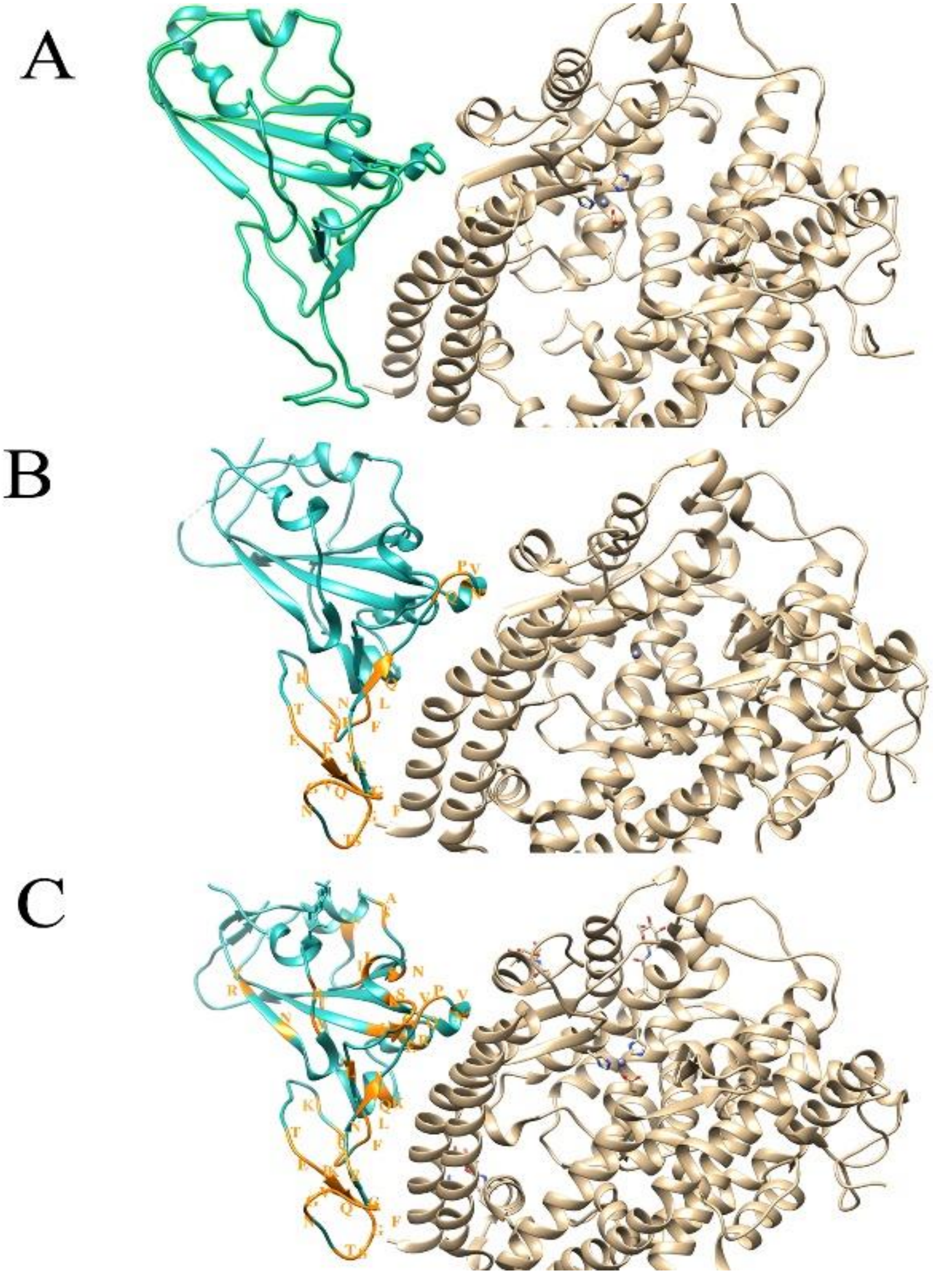

Figure S1. Structural representations for three structures of spike-ACE2 complexes: (A) SARS-CoV, (B) chimeric structure and (C) SARS-CoV-2. Mutations are highlighted for chimeric and SARS-CoV-2 structures. The images have been obtained by UCSF Chimera 1.13.1 (http://www.rbvi.ucsf.edu/chimera/)[47].

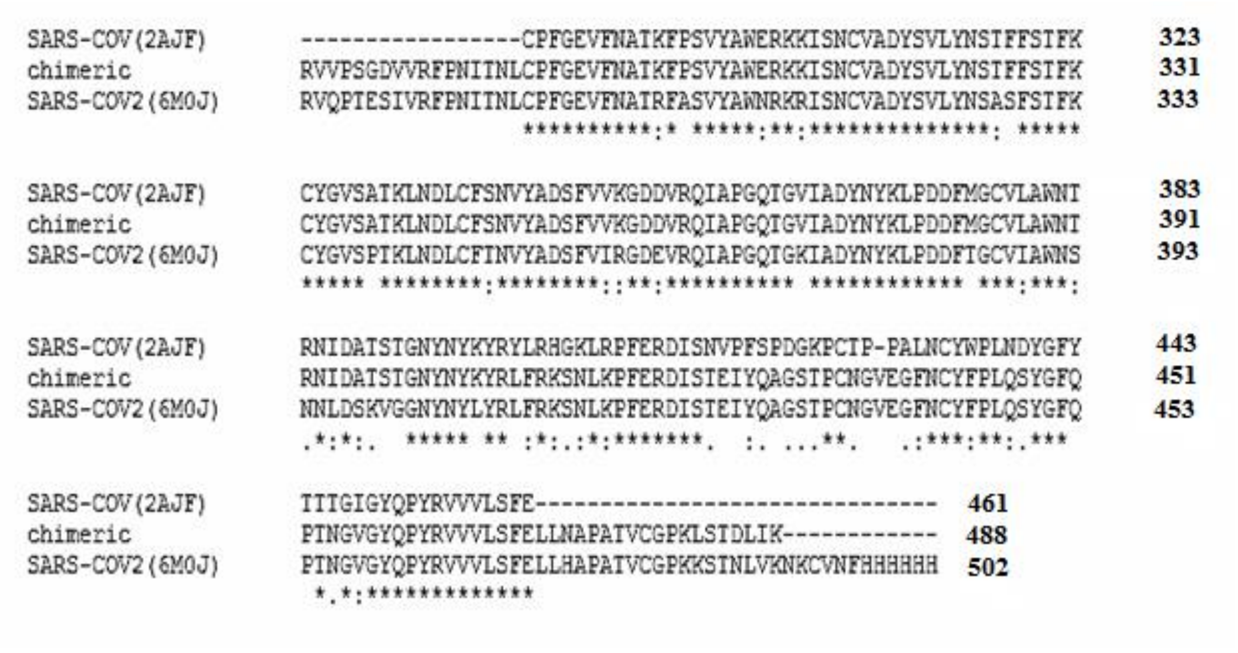

Figure S2. Sequence alignment of the spike proteins: SARS-CoV (2ajf), chimeric structure (6vw1) and SARS-CoV-2 (6m0j).

Table S1. List of amino acid mutations in receptor-binding motif of SARS-CoV-2 compared with SARS-CoV

SARS-CoV	SARS-CoV-2
Tyr442	Leu455
Leu443	Phe456
His445	Lys458
Gly446	Ser459
Lys447	Asn460
Arg449	Lys462
Asn457	Thr470
Val458	Glu471
Pro459	Ile472
Phe460	Tyr473
Ser461	Gln474
Pro462	Ala475
Asp 463	Gly476
Gly464	Ser477
Lys465	Thr478
Thr468	Asn481
Pro469	Gly482
Pro470	Glu484
Ala471	Gly485
Leu472	Phe486
Trp476	Phe490
Asn479	Gln493
Asp480	Ser494
Tyr484	Gln498
Thr485	Pro499
Thr487	Asn501
Ile 489	Val503

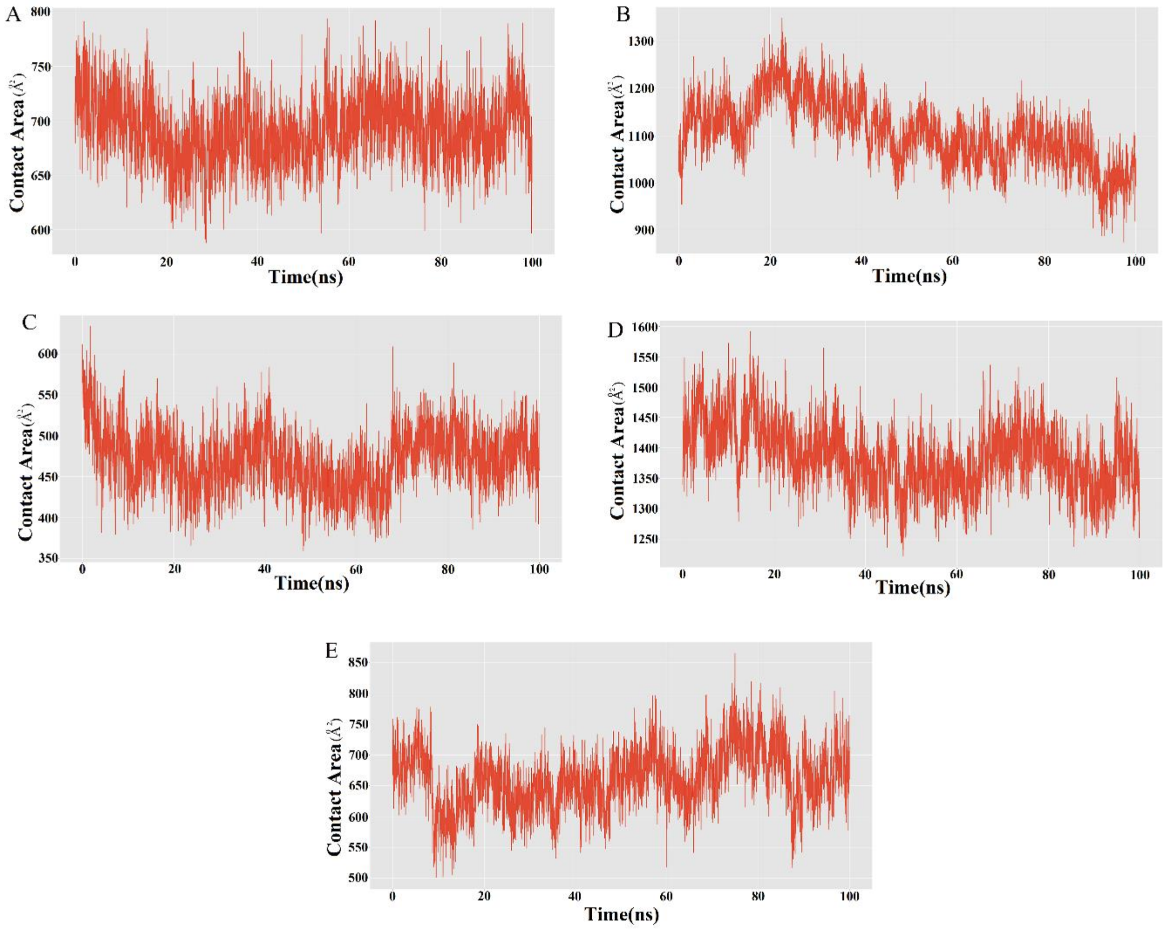

Figure S3. Contact area graphs for SARS-CoV-ACE2 complex (2ajf) during simulation. The contact areas between receptor-binding motif of SARS-CoV spike protein and different regions of ACE2 including residues 19-33 (A), 35-194 (B), 196-227 (C), 266-344 (D) and 541-616 (E). The images have been obtained by PyContact (https://pycontact.github.io/)[23].

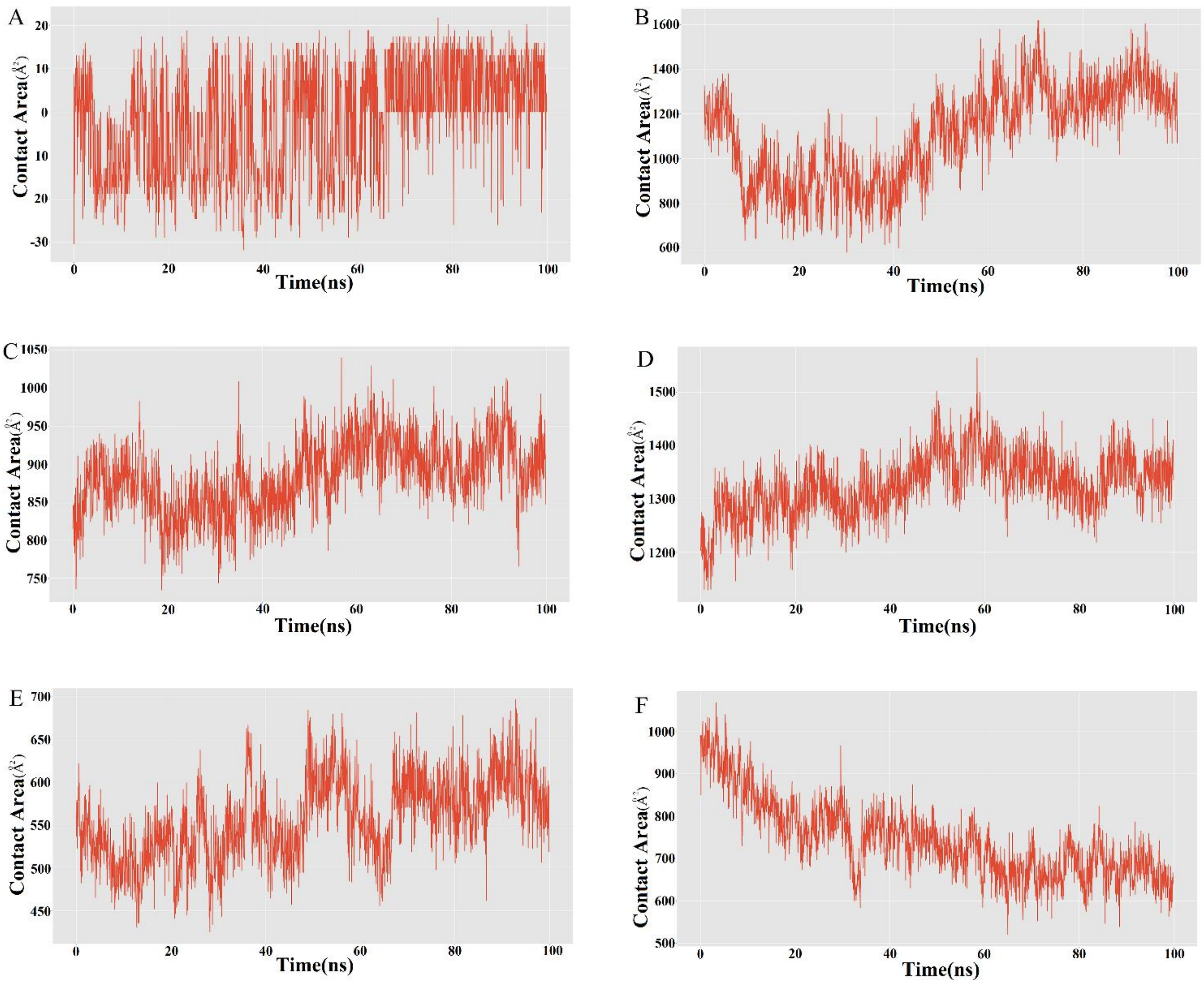

Figure S4. Contact area graphs for SARS-CoV-2-ACE2 complex (6m0j) during simulation. The contact areas between receptor-binding motif of SARS-CoV-2 spike protein and different regions of ACE2 including residues 19-33 (A), 35-194 (B), 242-264 (C), 266-344 (D), 346-372 (E) and 541-616 (F). The images have been obtained by PyContact (https://pycontact.github.io/)[23].

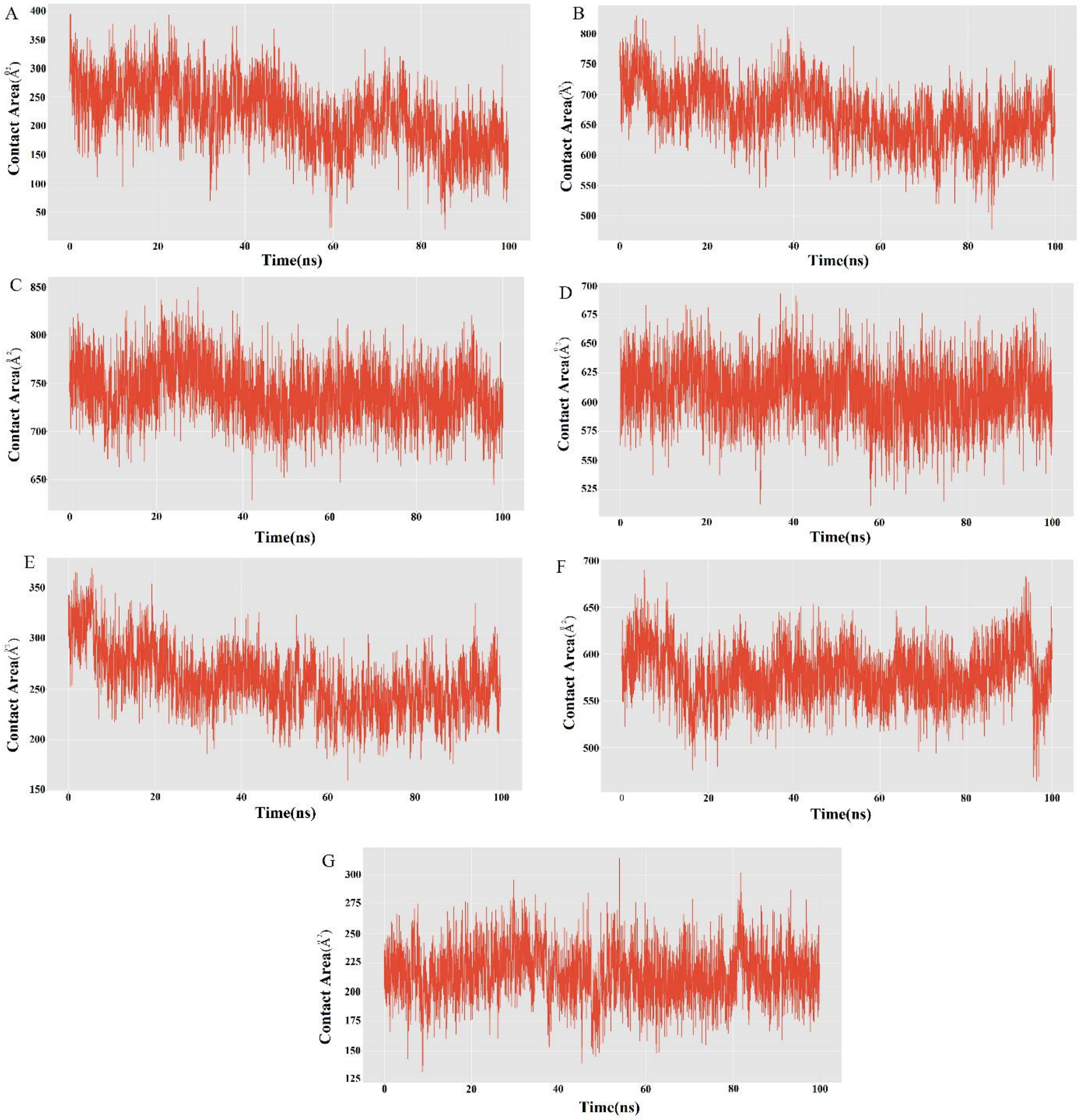

Figure S5. Contact area graphs for chimeric structure-ACE2 complex (6vw1) during simulation. The contact areas between receptor-binding motif of chimeric structure and different regions of ACE2 including residues 1-4 (A), 16-175 (B), 224-246 (C), 248-326 (D), 328-364 (E), 471-468 (F) and 523-596 (G). The images have been obtained by PyContact (https://pycontact.github.io/)[23].

Table S2. Free energy decomposition of the SARS-CoV residues in spike-ACE2 complex

Residue	Van der Waals	Electrostatic	Polar Solvation	Non-Polar Solv	TOTAL
Tyr440	-0.5155	0.0577	-0.0475	-0.0853	-0.0861±0.1095
Arg441	-0.06075	-94.0605	94.1639	0.0	0.04274±0.0100
Tyr442	-1.2877	-4.4947	4.5442	-0.4156	-1.6539±0.4785
Leu443	-0.55025	-0.2584	0.4032	-0.1094	-0.5149±0.0363
Arg444	0.0387	-87.2232	87.2925	0.0	0.0304±0.0070
His445	-0.0270	0.9190	-0.8239	0.0	0.0680±0.0041
Gly446	-0.0075	0.33444	-0.3127	0.0	0.01424±0.0020
Lys447	-0.0155	-93.0455	93.0662	0.0	0.00524±0.0126
Leu448	-0.0072	0.00024	0.00599	0.0	-0.00100±0.00079
Arg449	-0.0037	-74.6127	74.5985	0.0	-0.0180±0.0006
Pro450	-0.00200	-0.439249	0.4385	0.0	-0.002749±0.000892
Phe451	-0.00200	-0.695499	0.694750	0.0	-0.002749±0.00021
Glu452	-0.002999	78.6145	-78.583	0.0	0.028500±0.0055396
Arg453	-0.00400	-81.79400	81.773,2	0.0	-0.025000±0.003409
Asp454	-0.00625	88.57325	-88.497	0.0	0.069999±0.0046233
Ile455	-0.00500	0.62100	-0.610250	0.0	0.005750±0.0011
Ser456	-0.00675	1.797499	-1.7675	0.0	0.02325000±0.001138
Asn457	-0.0127	0.53275	-0.45125	0.0	0.0687499±0.01212628
VAL458	-0.01624	0.667250	0.2663	0.0	0.027500±0.00192
Pro459	-0.0384	-1.5322	1.5877	0.0	0.017000±0.0054
Phe460	-0.264750	0.847249	-0.598749	-0.0133379	-0.0295880±0.04822
Ser461	-0.17799	-2.074	2.33775	0.0	0857499±0.00397
Pro462	-2.07824	-3.249250	3.6332499	-0.3785706	-2.0728206±0.6382928
Asp463	-0.99375	94.6152	-93.225000	-0.1227563	0.27374359±0.153622
Gly464	-0.052499	-1.076499	1.1467500	0.0	0.01774999±0.015282

Lys465	-0.219500	-98.7687500	99.376	-0.0205073	0.36724259±0.1875600
Pro466	-0.021750	0.116749	-0.0952500	0.0	-0.0002500±0.0040369
Cyc467	-0.04899	-0.554749	0.6692499	0.0	0.065500±0.0008291
Thr468	-0.042500	0.066500	0.108499	0.0	0.132499±0.03570
Pro469	-0.15774	-2.17500	2.35325	0.0	0.020499±0.007652
Pro470	-0.748250	-0.29700	0.7505000	-0.177561	-0.47231100±0.45591
Ala471	-0.50225	1.0507499	-0.559499	-0.0628848	-0.07388480±0.181488
Leu472	-2.24925	-2.41200	3.10499	-0.5715666	-2.127816±0.30896
Asn473	-2.507749	-2.682500	4.24,0	-0.3706758	-1.320925±0.383652
CYS474	-0.306250	1.0912500	-0.6817500	-0.000239400	0.10301060±0.0312150
TYR475	-3.7625	-1.071500	3.24625	-0.6433883	-2.2311383±0.339181
TRP476	-0.2217500	-2.9794999	1.98324999	-0.0560771	-1.274077200±0.807167
PRO477	-0.18049	1.034999	-0.9515	0.0	-0.096999±0.0351158
LEU478	-0.273749	-1.33075	1.09525	-0.0177839	-0.5270339±0.32764
ASN479	-1.83100	-7.953499	8.044	-0.3598308	-2.10033079±0.7966147
ASP480	-1.02300	128.9555	-125.962	-0.190553	1.77994659±0.1701833
TYR481	-0.714249	-0.01500	0.75899999	-0.0959778	-0.0662277±0.0739066
GLY482	-0.96150	-0.4837499	0.9595	-0.1228518	-0.608601799±0.071494
PHE483	,-0.381500	-0.55724	0.685750	0.0	-0.2530±0.06141
TYR484	-3.42924	-6.82199	5.89849	-0.64378	-4.996536±0.813662
THR485	-0.39400	0.3257500	0.469249	-0.00118	0.399810±0.02827
THR486	-2.4535	-5.73374	5.51825	-0.788527	-3.4575277±0.423244
THR487	-2.80725	-3.038999	3.073499	-0.218493	-2.991242±0.178196
GLY488	-1.21624	-3.105499	2.88975	0.015074	-1.671684±0.08438
ILE489	-0.9	-1.33374	1.374	-0.236890	-1.0966408±0.26180
GLY490	-0.1640	-1.80075	1.991750	0.0	0.02699±0.02699
TYR491	-3.67949	-2.69575	4.369499	-0.57746	-2.58321±0.1613

GLN492	-0.37750	-3.75749	4.43325	-0.03858	0.259665±0.03482
PRO493	-0.062499	0.830249	-0.8345	0.0	-0.066750±0.0085

Table S3. Free energy decomposition of the SARS-CoV-2 residues in spike-ACE2 complex

Residue	Van der Waals	Electrostatic	Polar Solvation	Non-Polar Solv	TOTAL
Leu452	-0.0620	-0.9402	0.984	0.0	0.0174±0.0037
Tyr453	-0.9207	-0.5615	1.7677	-0.1430	0.1424±0.0960
Arg454	-0.0935	-94.970	95.1544	0.0	0.0900±0.0066
Leu455	-2.69	1.7307	-1.5465	-0.2712	-2.7769±0.17222
Phe456	-2.147	-0.29300	1.1042	-0.3387	-1.6744±0.2814
Arg457	-0.0742	-87.5895	87.7722	0.0	0.1084±0.0092
Lys458	-0.06699	-83.2865	83.4644	0.0	-0.11099±0.03423
Ser459	-0.0197	0.8854	-0.8342	0.0	0.0314±0.0028
Asn460	-0.0179	-1.4041	1.47375	0.0	-0.04425±0.0170
Lus461	-0.011	0.38074	-0.3777	0.0	-0.0079±0.0023
Lys462	-0.0044	-78.2182	78.2075	0.0	-0.0152±0.00143
Pro463	-0.0033	-0.6742	0.6729	0.0	-0.0042±0.0002
Phe464	-0.003	-0.9977	0.997	0.0	-0.0037±0.00021
Glu465	-0.00425	82.993	-82.93875	0.0	0.04999±0.00372
Arg466	-0.00574	-83.85675	83.8375	0.0	-0.02499±0.001499
Asp467	-0.00999	89.5095	-89.42725	0.0	0.07224999±0.006493
Ile468	-0.008249	0.5979999	-0.57775	0.0	0.012000±0.001274
Ser469	-0.009249	1.7684999	-1.73775	0.0	0.0214999±0.0046165
Thr470	-0.01900	0.261249	-0.1747499	0.0	0.0674999±0.007462
Glu471	-0.02474	85.77875	-85.59025	0.0	0.163750±0.009429
Ile472	-0.0814999	-1.06925000	1.1137499	0.0	-0.0369999±0.0070799
Tyr473	-0.751500	-0.7197500	1.3787500	-0.103559	-0.1960594±0.0736
Gln474	-0.374500	-2.604999	3.4457500	0.0	0.4662500±0.014006

Ala475	-2.4	-5.4	0.93	0.0	-6.86±0.346735
Gly476	-1.33	-0.819	0.210	0.0	-1.941±-0.5075
SER477	-0.2575	-1.019	0.236	0.0	-1.0412±0.285
THR478	-0.235	-0.319	0.2847	0.0	-0.27024±0.099
PRO479	-0.0482	2.0280	-0.471	0.0	1.5087±0.080
CYS480	-0.0392	-0.10	0.02849	0.0	-0.1107±0.485405
ASN481	-0.02050	-1.5867	0.36874	0.0	-1.2385±0.2653
GLY482 insertion	-0.0130	-0.2397	0.06024	0.0	-0.1924±0.0615
VAL483	-0.0657	-0.7180	0.15175	0.0	-0.6320±0.13198
GLU484	-0.2472	94.5339	-20.826	0.0	73.46074±2.36857
GLY485	-0.8525,	-0.9514	0.3054	0.0	-1.49850±0.1309
PHE486	-4.986	-2.78225	0.95475	0.0	-6.81350±0.3228268
ASN487	-2.16875	-4.966999	4.66175	-0.1221767	-2.596176±0.462978
CYS488	-0.40399	0.468250	-0.00749	0.0	0.0567500±0.045828
TYR489	-4.4825	-2.253250	3.984	-0.6036408	-3.355390±0.159110
PHE490	-0.70149	0.88350	-0.406500	-0.05899	-0.2834949±0.28366
PRO491	-0.1750	1.77700	-1.7545	0.0	-0.152499±0.027978
LEU492	-0.2450	-1.16200	1.186499	-0.0053496	-0.225849±0.304708
GLN493	-2.54999	-13.47625	12.46575	-0.5655132	-4.126013±0.46159
SER494	-0.35450	2.19399	-1.487500	-0.0140579	0.33794±0.02797
TYR495	-0.411249	-0.23124	0.905999	-0.000637	0.262862±0.010493
GLY496	-1.247	-4.69324	2.67925	-0.213111	-3.47411±0.334680
PHE497	-0.41799	0.014250	0.34525	0.0	-0.05849±0.07135
GLN498	-1.38974	-8.909749	6.52150	-0.4337334	-4.211733±0.50664
PRO499	-0.43949	1.74700	-1.365	-0.035546	-0.093046±0.0411
THR500	-3.15799	-1.998249	3.2177499	-0.711802	-2.650302±0.68225
ASN501	-3.316,0	-3.60650	4.4354999	-0.189327	-2.6763275±0.52674

**GLY502**	**-1.3195**	**-3.22649**	**2.99075**	**-0.30320**	**-1.8584581±0.05986**
**VAL503**	**-0.74**	**-1.45700**	**1.67075**	**-0.1462068**	**-0.6724567±0.19391**
**GLY504**	**-0.23475**	**-1.84649**	**2.08875**	**-0.002628**	**0.0048720±0.02468**
**TYR505**	**-4.3575**	**-2.80150**	**4.218**	**-0.744759**	**-3.6857590±0.3645386**
**GLN506**	**-0.525249**	**-3.357499**	**3.85425**	**-0.09606**	**-0.1245623±0.3135**
**PRO507**	**-0.07099**	**0.815250**	**-0.857500**	**0.0**	**-0.11324±0.00537**
**TYR508**	**-0.055750**	**-0.243749**	**0.30950**	**0.0**	**0.0100±0.00791**

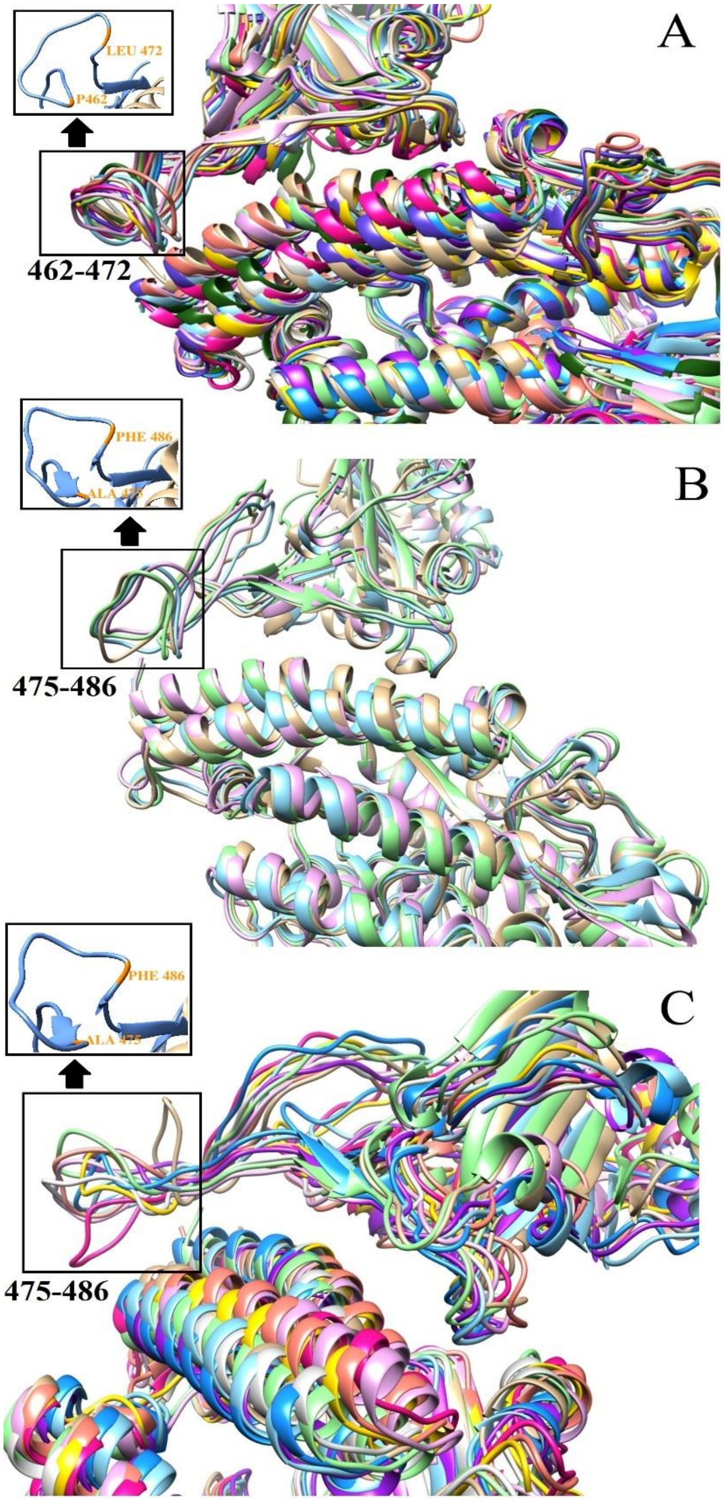

Figure S6. Comparison of proteins structures during simulation (with more focus on the binding area): (A) SARS-CoV, (B) chimeric structure and (C) SARS-CoV-2, the most fluctuated regions are located at residues 475-486 for SARS-COV2 and 462-472 for SARS-CoV. The images have been obtained by UCSF Chimera 1.13.1 (http://www.rbvi.ucsf.edu/chimera/)[47].

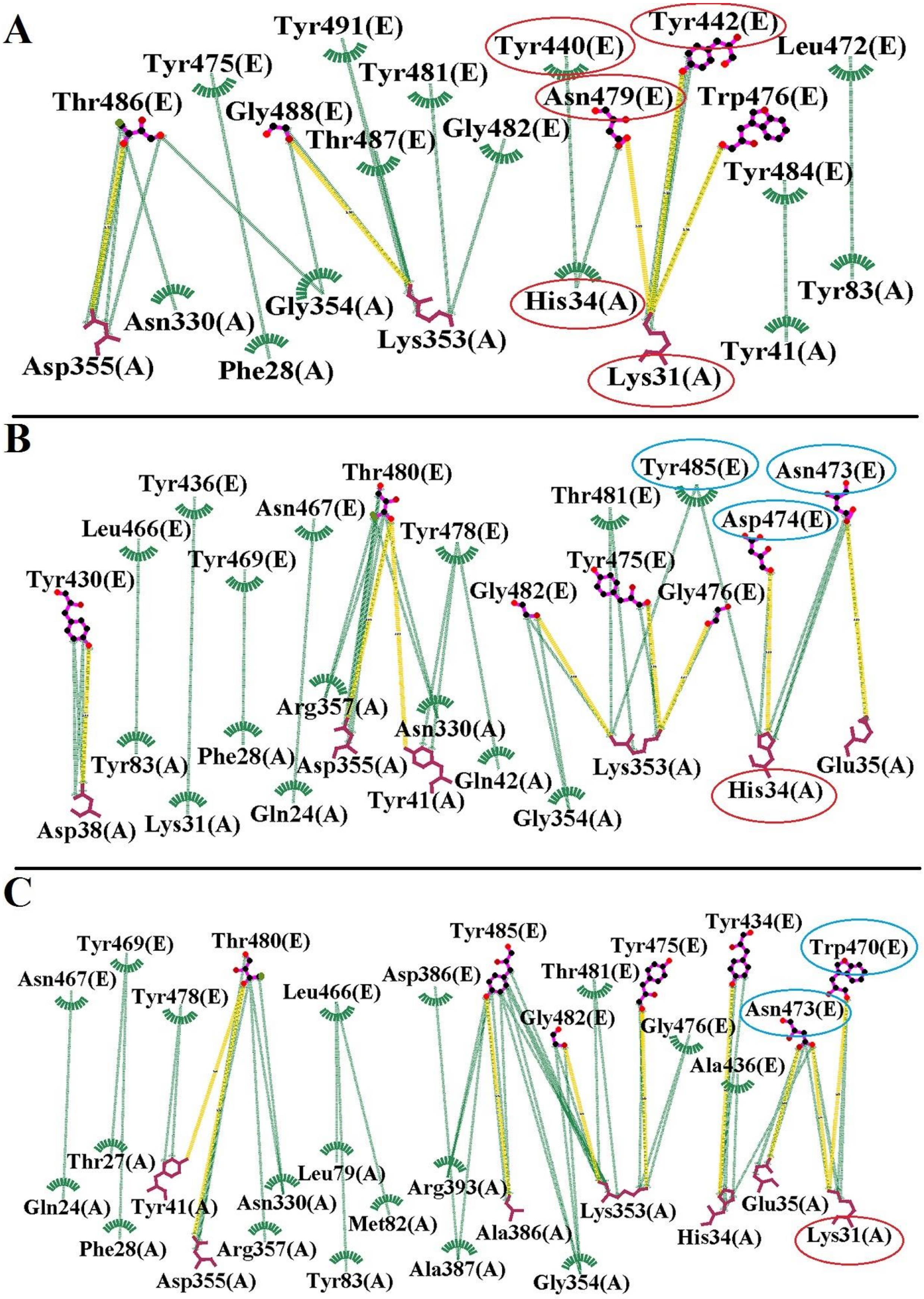
A
Tyr491(E)
Tyr440(E)
Tyr442(E)
Leu472(E)
Tyr475(E)
Tyr481(E)
Asn479(E)
Trp476(E)
Thr486(E)
Gly488(E)
Thr487(E)
Gly482(E)
Tyr484(E)
Gly354(A)
His34(A)
Tyr83(A)
Asn330(A)
Lys353(A)
Tyr41(A)
Asp355(A)
Phe28(A)
Lys31(A)
B
Tyr485(E)
Asn473(E)
Thr480(E)
Tyr436(E)
Thr481(E)
Asn467(E)
Asp474(E)
Leu466(E)
Tyr478(E)
Tyr469(E)
Tyr475(E)
Tyr430(E)
Gly482(E)
Gly476(E)
Arg357(A)
Asn330(A)
Tyr83(A)
Phe28(A)
Asp355(A)
Lys353(A)
Glu35(A)
Gln42(A)
Tyr41(A)
His34(A)
Asp38(A)
Lys31(A)
Gln24(A)
Gly354(A)
C
Tyr434(E)
Tyr469(E)
Thr480(E)
Tyr485(E)
Tyr475(E)
Trp470(E)
Asn467(E)
Asp386(E)
Thr481(E)
Tyr478(E)
Leu466(E)
Gly482(E)
Gly476(E)
Asn473(E)
Ala436(E)
Thr27(A)
Leu79(A)
Arg393(A)
Gln24(A)
Tyr41(A)
Asn330(A)
Lys353(A)
Glu35(A)
Met82(A)
Ala386(A)
His34(A)
Lys31(A)
Phe28(A)
Arg357(A)
Ala387(A)
Asp355(A)
Tyr83(A)
Gly354(A)

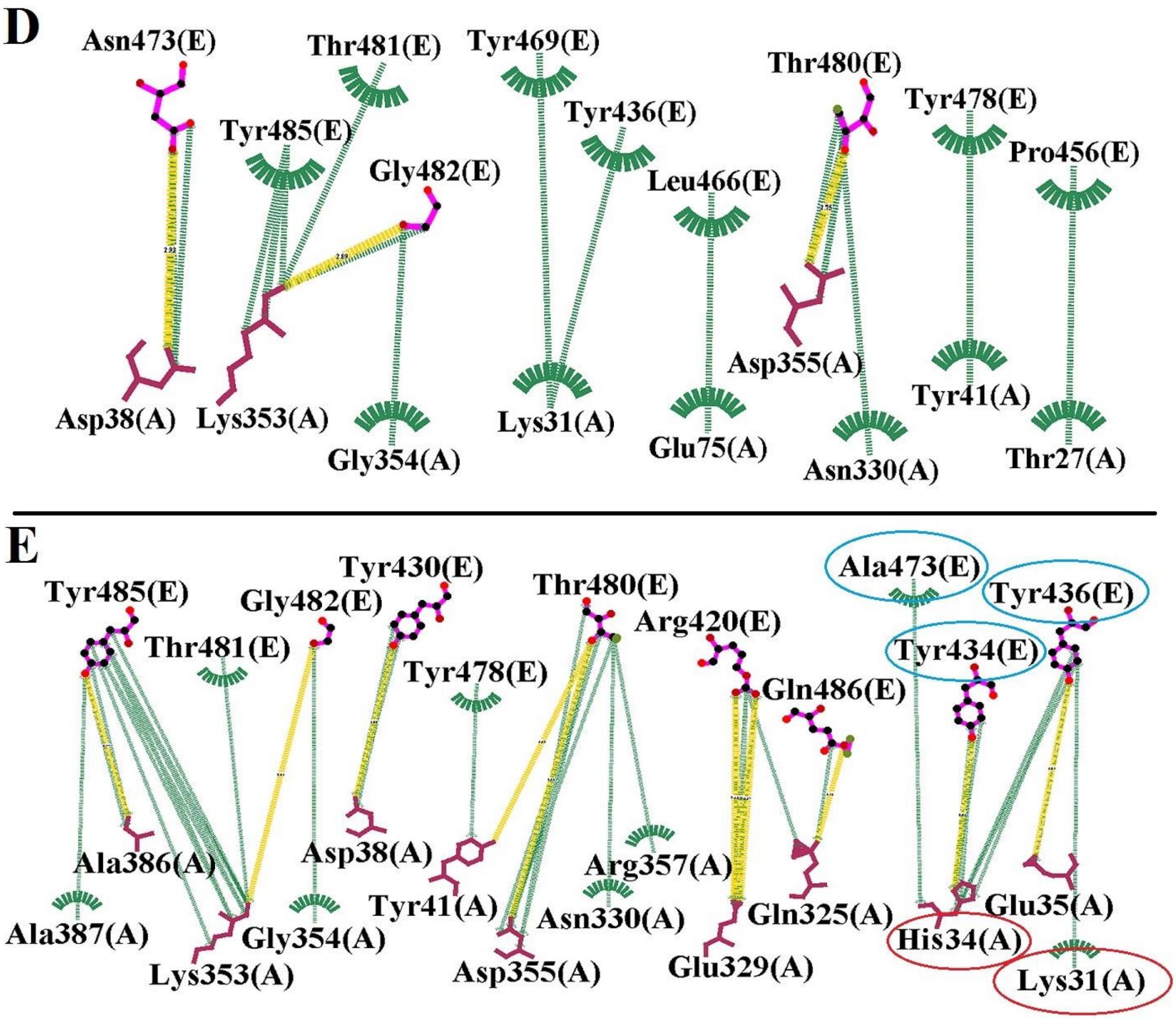

Figure S7. Interaction schemes of the selected alanine scanning in SARS-CoV-ACE2 complex: (A) is native structure of SARS-CoV and (C, D and E) are alanine substitutions for residues Tyr440, Tyr442, Leu443, and Asn479 respectively. The A and E in the parenthesis after residue names denote chains A (ACE2) and E (SARS-CoV). Also, hydrogen bonds and hydrophobic interactions are colored in yellow and green lines respectively. The red circles in part A represent the critical interactions in the native st ructure of SARS-CoV-ACE2 complex. The red and blue circles in parts B, C, D and E represent those interactions which remained intact or altered after alanine scanning (respectively). The images have been obtained by LigPlot^{+} v.1.4.5 (https://www.ebi.ac.uk/thornton-srv/software/LigPlus/)[22].

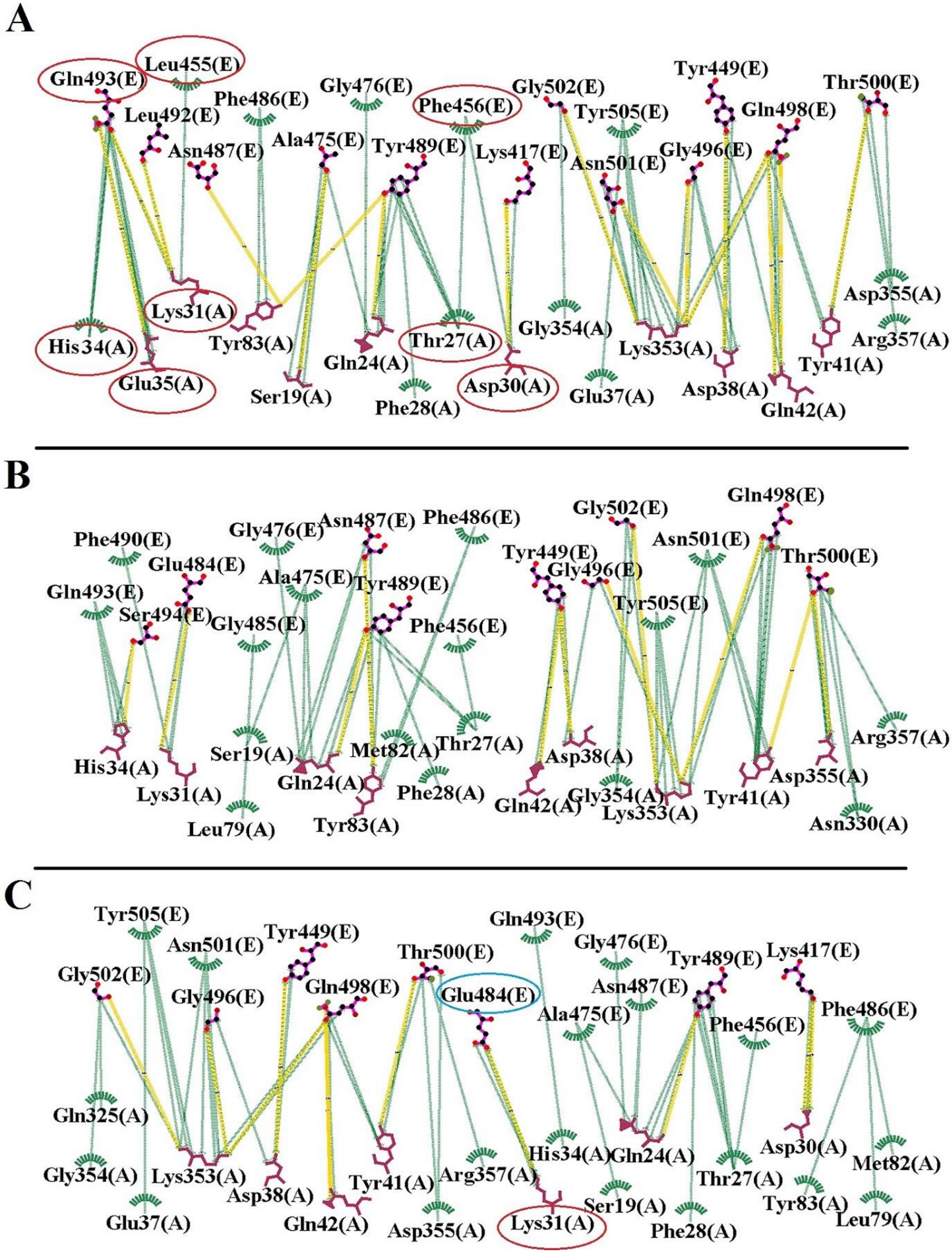

A
Gln493(E)
Leu455(E)
Phe486(E)
Gly476(E)
Phe456(E)
Gly502(E)
Tyr449(E)
Thr500(E)
Leu492(E)
Tyr505(E)
Gln498(E)
Asn487(E)
Ala475(E)
Tyr489(E)
Lys417(E)
Asn501(E)
Gly496(E)
Asp355(A)
Lys31(A)
Gly354(A)
Arg357(A)
His34(A)
Tyr83(A)
Gln24(A)
Thr27(A)
Lys353(A)
Tyr41(A)
Glu35(A)
Ser19(A)
Asp30(A)
Glu37(A)
Asp38(A)
Phe28(A)
Gln42(A)
B
Gly502(E)
Gln498(E)
Phe486(E)
Gly476(E)
Asn487(E)
Phe490(E)
Asn501(E)
Tyr449(E)
Thr500(E)
Glu484(E)
Gly496(E)
Ala475(E)
Tyr489(E)
Gln493(E)
Tyr505(E)
Ser494(E)
Gly485(E)
Phe456(E)
Arg357(A)
Thr27(A)
Met82(A)
Ser19(A)
His34(A)
Asp38(A)
Asp355(A)
Gln24(A)
Gly354(A)
Tyr41(A)
Lys31(A)
Phe28(A)
Gln42(A)
Lys353(A)
Asn330(A)
Leu79(A)
Tyr83(A)
C
Tyr505(E)
Gln493(E)
Tyr449(E)
Gly476(E)
Asn501(E)
Lys417(E)
Thr500(E)
Tyr489(E)
Gly502(E)
Gln498(E)
Asn487(E)
Gly496(E)
Glu484(E)
Phe486(E)
Ala475(E)
Phe456(E)
Gln325(A)
His34(A)
Gln24(A)
Asp30(A)
Gly354(A)
Lys353(A)
Arg357(A)
Thr27(A)
Met82(A)
Tyr41(A)
Asp38(A)
Ser19(A)
Tyr83(A)
Glu37(A)
Gln42(A)
Leu79(A)
Asp355(A)
Lys31(A)
Phe28(A)

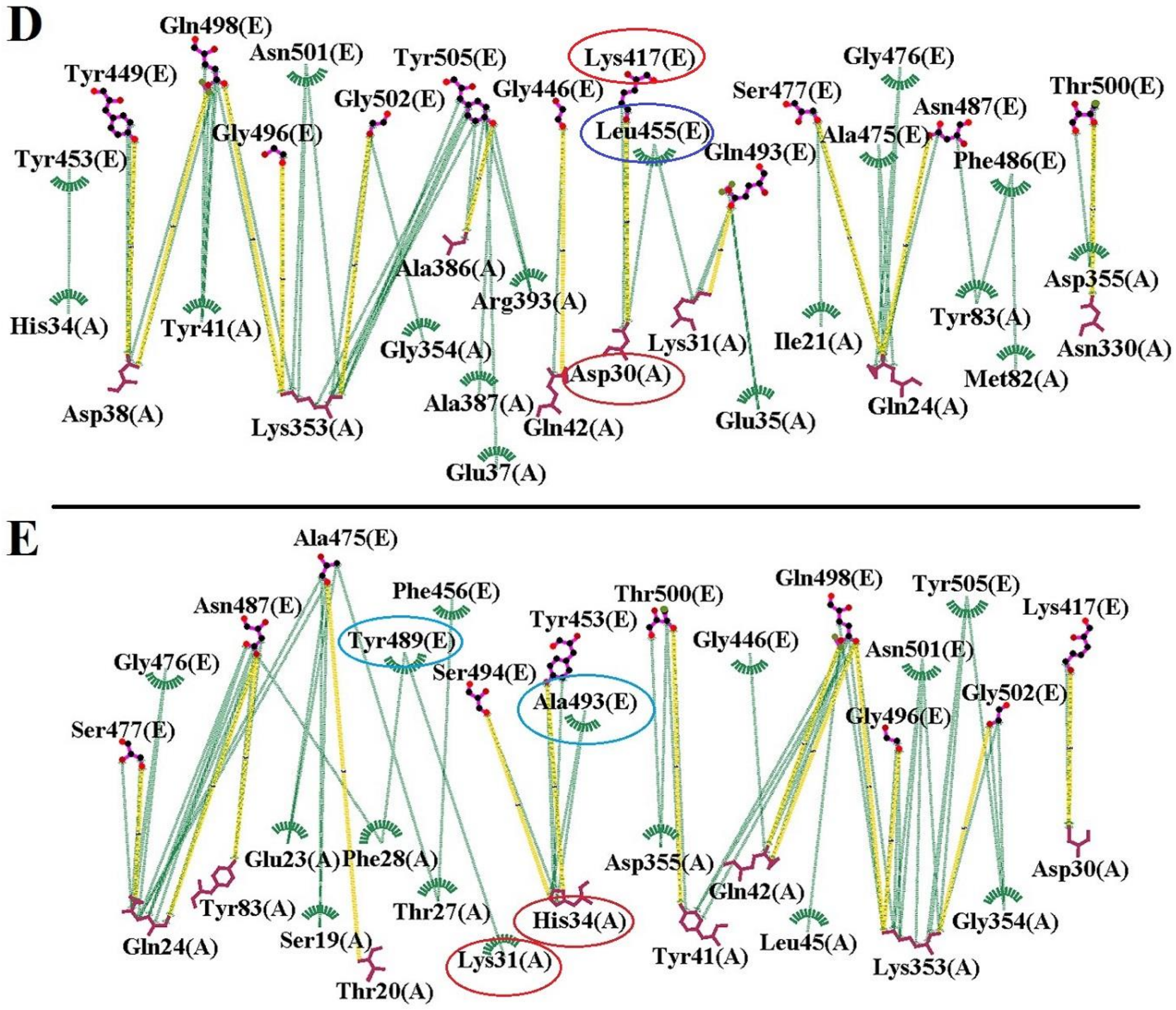

Figure S8. Interaction schemes for the selected alanine scanning in SARS-CoV-2-ACE2 complex: (A) is native structure of SARS-CoV-2 and (C, D and E) are alanine substitutions for residues Tyr453, Leu455, Phe456, and Gln493 respectively. The A and E in the parenthesis after residue names denote chains A (ACE2) and E (SARS-CoV-2). Also, hydrogen bonds and hydrophobic interactions are colored in yellow and green lines respectively. The red circles in part A indicate the critical interactions in the native structure of SARS-CoV-2-ACE2 complex. The red and blue circles in parts B, C, D and E represent those interactions which remained intact or altered after alanine scanning (respectively). The images have been obtained by LigPlot^{+} v.1.4.5 (https://www.ebi.ac.uk/thornton-srv/software/LigPlus/)[22].
